# Dhurrin metabolism in the developing grain of *Sorghum bicolor* (L.) Moench investigated by metabolite profiling and novel clustering analyses of time-resolved transcriptomic data

**DOI:** 10.1186/s12864-016-3360-4

**Published:** 2016-12-13

**Authors:** Lasse Janniche Nielsen, Peter Stuart, Martina Pičmanová, Simon Rasmussen, Carl Erik Olsen, Jesper Harholt, Birger Lindberg Møller, Nanna Bjarnholt

**Affiliations:** 1 Plant Biochemistry Laboratory, Department of Plant and Environmental Sciences, University of Copenhagen, Thorvaldsensvej 40, Frederiksberg C, 1871 Denmark; 2 VILLUM Research Center for Plant Plasticity, University of Copenhagen, Thorvaldsensvej 40, Frederiksberg C, 1871 Denmark; 3 Seedtek, 12 Kestrel Court, Toowoomba, 4350 Australia; 4 Center for Synthetic Biology ‘bioSYNergy’, University of Copenhagen, Copenhagen, Denmark; 5 Department of Systems Biology, Technical University of Denmark, Kemitorvet, 2800 Kgs. Lyngby, Denmark; 6 Carlsberg Research Laboratory, J.C. Jacobsens Gade 4, 1799 Copenhagen V, Denmark; 7 Novozymes A/S, Krogshoejvej 36, 2880 Bagsvaerd, Denmark

**Keywords:** Sorghum, Endogenous turnover, Transcriptome analysis, UDP-glycosyltransferase, Glutathione S-transferase, Proanthocyanidins

## Abstract

**Background:**

The important cereal crop *Sorghum bicolor* (L.) Moench biosynthesize and accumulate the defensive compound dhurrin during development. Previous work has suggested multiple roles for the compound including a function as nitrogen storage/buffer. Crucial for this function is the endogenous turnover of dhurrin for which putative pathways have been suggested but not confirmed.

**Results:**

In this study, the biosynthesis and endogenous turnover of dhurrin in the developing sorghum grain was studied by metabolite profiling and time-resolved transcriptome analyses. Dhurrin was found to accumulate in the early phase of grain development reaching maximum amounts 25 days after pollination. During the subsequent maturation period, the dhurrin content was turned over, resulting in only negligible residual dhurrin amounts in the mature grain. Dhurrin accumulation correlated with the transcript abundance of the three genes involved in biosynthesis. Despite the accumulation of dhurrin, the grains were acyanogenic as demonstrated by the lack of hydrogen cyanide release from macerated grain tissue and by the absence of transcripts encoding dhurrinases. With the missing activity of dhurrinases, the decrease in dhurrin content in the course of grain maturation represents the operation of hitherto uncharacterized endogenous dhurrin turnover pathways. Evidence for the operation of two such pathways was obtained by metabolite profiling and time-resolved transcriptome analysis. By combining cluster- and phylogenetic analyses with the metabolite profiling, potential gene candidates of glutathione S-transferases, nitrilases and glycosyl transferases involved in these pathways were identified. The absence of dhurrin in the mature grain was replaced by a high content of proanthocyanidins. Cluster- and phylogenetic analyses coupled with metabolite profiling, identified gene candidates involved in proanthocyanidin biosynthesis in sorghum.

**Conclusions:**

The results presented in this article reveal the existence of two endogenous dhurrin turnover pathways in sorghum, identify genes putatively involved in these transformations and show that dhurrin in addition to its insect deterrent properties may serve as a storage form of reduced nitrogen. In the course of sorghum grain maturation, proanthocyanidins replace dhurrin as a defense compound. The lack of cyanogenesis in the developing sorghum grain renders this a unique experimental system to study CNglc synthesis as well as endogenous turnover.

**Electronic supplementary material:**

The online version of this article (doi:10.1186/s12864-016-3360-4) contains supplementary material, which is available to authorized users.

## Background

Cyanogenic glycosides (CNglcs) are bioactive specialized metabolites found in more than 3,000 plant species distributed in 130 different plant families of angiosperms, gymnosperms and ferns [1] with a large proportion found among of our cultivated plants [2]. Examples of cyanogenic crop plants include barley (*Hordeum vulgare*) [3], wheat (*Triticum aestivum*) [2, 4], sorghum (*Sorghum bicolor* (L.) Moench) [5], cassava (*Manihot esculenta*) [6], almond (*Prunus dulcis*) [7], cherry (*Prunus spp.*) [8] and apple (*Malus pumilia* hybrids) [9]. Upon tissue disruption, the CNglcs stored in the tissues of cyanogenic plants, are brought in contact with endogenous β-glucosidases (BGDs) resulting in hydrolysis and release of the cyanohydrin aglycone, which spontaneously or catalyzed by an α-hydroxynitrile lyase (HNL) dissociates to release hydrogen cyanide (HCN) and a keto compound. This pathway is often referred to as the bioactivation pathway and the process as cyanogenesis [10]. The toxic effect of HCN is caused by its ability to inhibit metalloenzymes and in particular cytochrome *c* oxidase, the key enzyme in the respiratory electron transport chain in mitochondria [11]. CNglc storage in plants and the release of HCN upon tissue disruption may serve as a deterrent against generalist herbivores [12, 13]. In addition to herbivore defense, CNglcs may serve a multiplicity of other biological functions [1, 14].

In the rubber tree (*Hevea brasiliensis*), linamarin accumulates in the mature seed [15]. Upon germination, the CNglc is converted into the cyanogenic diglucoside (CNdglc) linustatin and transported to the cotyledon where it is turned over [15, 16]. A similar situation has been observed in bitter almonds where prunasin is biosynthesized in the tegument of the almond fruit throughout development and eventually transported into the developing cotyledons of the kernel where it is converted into the CNdglc amygdalin [7]. Upon germination, the amygdalin stored in the cotyledon of the bitter almond variants is most likely catabolized as observed for amygdalin accumulated in seeds of black cherry (*Prunus serotina* Ehrh.) [17]. In sorghum, dhurrin derived CNdglcs are found in guttation droplets and are similarly hypothesized to act as transport forms [18]. Three CNdglcs which differ from each other in the configuration of the glucosidic linkage between the primary and secondary glucose molecule have been found [19], but their specific roles as possible transporters remain unknown.

In young sorghum seedlings, the CNglc dhurrin rapidly accumulated following germination after which biosynthesis decreased and turnover increased to result in a reduced concentration of dhurrin [20, 21]. This turnover of CNglcs was earlier thought to occur *via* the bio-activation pathway with the HCN released being incorporated into β-cyanoalanine by β-cyanoalanine synthase (CAS) and finally converted by nitrilases belonging to the NIT4 family to asparagine and to aspartate with concomitant release of ammonia [22, 23]. However, evidence for the occurrence of alternative turnover pathways that bypasses the release of the toxic intermediates HCN and β-cyanoalanine has been proposed [19, 24]. In the first alternative pathway suggested for sorghum, dhurrin was hypothesized converted to *p*-hydroxyphenylacetonitrile (pOHPCN) via an unknown novel BGD/protein co-factor complex [24]. No evidence has subsequently been found to support the presence of such a protein complex. Instead, more recent results pointed to the formation of pOHPCN through the action of glutathione S-transferases (GSTs) supported by genome wide association analysis of a sorghum population with variable leaf dhurrin levels, where a GST was shown to be associated with dhurrin concentration in leaves [25]. The pOHPCN was subsequently hydrolyzed to *p*-hydroxyphenylacetic acid (pOHPAAc) and ammonia by the action of a heteromeric enzyme complex composed of the enzymes NIT4A and NIT4B2 [24]. Finally, the pOHPAAc was glucosylated to produce *p*-glucosyloxyphenylacetic acid (pGlcPAAc) which has been shown to accumulate in vegetative tissue of different sorghum cultivars as the plants got older and the dhurrin content decreased [19, 24, 26]. The second proposed endogenous turnover pathway included a unifying set of proposed turnover products in sorghum, almond and cassava where the nitrile group of the CNglc is converted into an amide, a carboxylic acid and finally completely lost in a decarboxylation reaction [19]. For clarity, these possible intermediates are referred to as CNglc amide, CNglc acid and CNglc anitrile in this manuscript.

For investigating the turnover of CNglcs in cyanogenic plants, the developing grains of sorghum were selected as an experimental model system. Developing seeds are terminal sink organs and do not have net export of nutrients and amino acids [27] and therefore offer an attractive system for study of endogenous turnover processes. The mature sorghum grain contains only minute amounts or no dhurrin at all [4, 28, 29]. In this study, we report that dhurrin is formed in the initial stages of grain development and turned over in the course of grain maturation. The grain was found not to possess dhurrinase (DHR) activity signifying the operation of one or more endogenous turnover pathways not involving the release of toxic HCN. High concentrations of condensed tannins (proanthocyanidins) were formed throughout development and accumulated in the mature grain. For the investigation of endogenous turnover of dhurrin, time-resolved transcriptomic analyses guided the identification of novel gene candidates encoding enzymes belonging to the families of GSTs, the nitrilase superfamily and UDP-glycosyltransferases (UGTs). Based on the expression profiles of the genes supposedly involved in endogenous dhurrin turnover and the dhurrin metabolites formed, we propose a dual role for dhurrin in the developing grain of sorghum.

## Results

### Metabolite profiling of developing sorghum grain

**Fig. 1:**
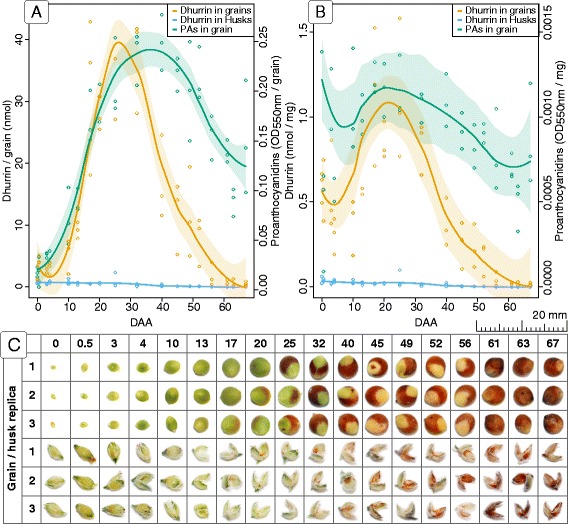
Concentrations of dhurrin and proanthocyanidins in developing grains. The concentration of dhurrin and proanthocyanidins (PAs) measured pr. grain (**a**) and pr. mg grain tissue (**b**) at different days after anthesis (DAA). The dhurrin concentration is measured in nmol and the PA concentration as absorbance. The *colored circles* represent the actual measurements in the three replicas and the *solid line* the trend line calculated using local polynomial regression fitting, with the area surrounding the average line representing the 95% confidence interval. Each measurement represents the average of 5 grains with one of the five grains and husks displayed in the table (**c**)

Immediately following anthesis, the total content of dhurrin in the grain was low and barely detectable (<2 nmol/grain, corresponding to 0.5 nmol/mg)). Dhurrin content then rapidly increased reaching a maximum 25 days after anthesis (DAA) (40 nmol/grain, 1.1 nmol/mg) (Fig. 1a and b). After this maximum, a similarly rapid decrease occurred which resulted in complete disappearance of dhurrin at maturity, 67 DAA (0 nmol/grain). In the same time period, the dhurrin content of the husk remained constant at 0.5 nmol/husk (0.02 nmol/mg). Accordingly, the concentration of dhurrin was approximately 25 times higher in the grain compared to the husk right after anthesis and 55 times higher at the maximum dhurrin concentration.

The presence of dhurrin turnover products in the extracts of the sorghum grain from anthesis to maturation was analyzed by LC-MS/MS. Dhurrin amide, dhurrin acid, pGlcPAAc and the dhurrin glucosides A, B and C were all found in the grain extracts. A previous study by Pičmanová et al. [19] detected the presence of caffeoyl-dhurrin (m/z: 496 [M + Na]^+^) and caffeoyl-dhurrin-acid (m/z: 515 [M + Na]^+^) in sorghum leaf extracts. These compounds were not found in the sorghum grain.

**Fig. 2:**
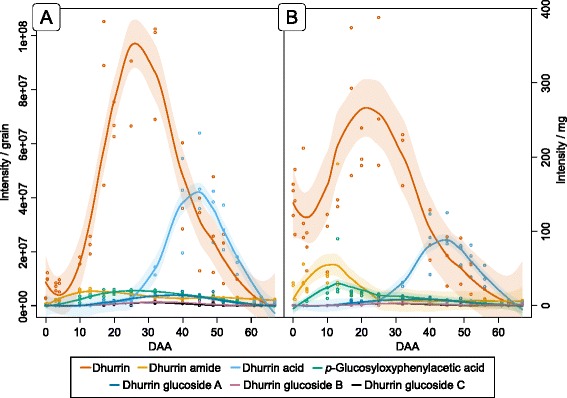
The accumulation of putative dhurrin turnover products. The concentration of dhurrin and putative turnover products measured as intensity pr. grain (**a**) and intensity pr. mg grain tissue (**b**) throughout development. The *colored circles* represent the actual measurements in the three replicates and the *solid line* the trend line calculated using local polynomial regression fitting. The area surrounding the average line representing the 95% confidence interval. The different colors corresponds to the different metabolites listed in the *box* below the graphs

The appearance of the putative dhurrin turnover products in the sorghum grain from anthesis to maturity in relation to the total content and concentration of dhurrin is shown in Fig. 2. In whole grains, dhurrin amide peaked around 10 DAA, i.e. immediately before the concentration of dhurrin started to increase. In contrast, the related compound dhurrin acid (formed either directly from dhurrin or via hydrolysis of the dhurrin amide), began to accumulate around 25 DAA, i.e. at the time point where the dhurrin content started to decrease. In relative terms, the dhurrin acid reached 34% of the total dhurrin concentration (values obtained by integrating the curves in Fig. 2), while dhurrin amide only reached 9%. Similarly, the content of pGlcPAAc reached 8% of the total dhurrin content. The dhurrin glucosides were observed to reach their maximum content at 32 DAA, i.e. a week after the dhurrin content started to decrease and reached 6% of the total dhurrin content.

### Proanthocyanidin content in the sorghum grain and husk from anthesis to maturity

A high concentration of proanthocyanidins (PAs) in developing and mature sorghum grains could increase herbivore resistance and compensate for the lack of dhurrin at maturity [30]. The concentration of PAs throughout grain development was therefore investigated. As part of the maturation process, the grain changed color from an initial green to a deep dark/brown color. The browning process was initiated 20 DAA (Fig. 1, panel c) and was correlated with formation of PAs (Fig. 1, panels a and b). In the sorghum grain, the content of PA increased from a low initial level to a maximum content per grain at 40 DAA. From 40 to 67 DAA, the content decreased to approximately half the maximum value. When measured per mg grain tissue, the variations in concentration of PAs was less pronounced with only a slight increased concentration around 20 DAA followed by a small decrease towards maturation.

### Cyanide potential of seeds

**Fig. 3:**
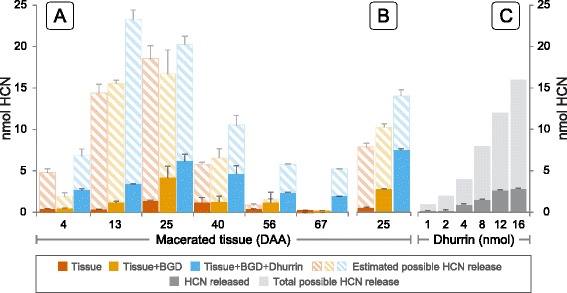
Cyanide potential in grains. nmol Hydrogen cyanide (HCN) released from macerated tissue at different developmental stages (**a**). The HCN release was measured from macerated tissue in buffer alone (Tissue, *red bar*), tissue in buffer plus 0.67 U/mL almond β-glucosidase (Tissue + BGD, *orange bar*) and tissue in buffer plus 0.67 U/mL almond β-glucosidase and an additional 5 nmol dhurrin (Tissue + BGD + Dhurrin, *blue bar*). For each sample/bar, the total possible release of HCN from dhurrin is indicated by the striped bar stacked on top of the measured HCN release. The total possible release of HCN is calculated from the equation describing the trendline for dhurrin in Fig. 1b. The HCN release was measured a second time with macerated tissue from 25 DAA with 5 U/mL almond β-glucosidase instead of 0.67 U/mL (**b**). As a control, an assay measuring HCN release from varying concentrations of dhurrin (1–16 nmol) in buffer was also performed (**c**)

The total content of a CNglc in plant tissue is often referred to as the cyanide potential (HCNp). Despite incubation for 2.5 h following maceration, only a small fraction of the total amount of dhurrin present in the samples as determined by LC-MS/MS analysis was hydrolyzed in the native samples (Fig. 3a). The amount of dhurrin hydrolyzed is positively correlated with the total estimated amount of dhurrin but does not exceed the amount undergoing non-enzymatic hydrolysis in the same time frame (Fig. 3c). Addition of almond BGD to homogenates of the initial samples (4–25 DAA), increased the amount of HCN released and was positively correlated to the total dhurrin content although complete hydrolysis of the dhurrin present was not observed. From 40 to 67 DAA, the addition of BGD did not increase the amount of HCN released. Spiking of the samples with 5 nmol dhurrin increased the release of HCN slightly, but did not result in an additional 5 nmol HCN compared to the HCN released from the samples containing tissue plus almond BGD. Increasing the concentration of BGD from 0.67 to 5U/mL only increased the HCN release relative to the total dhurrin content in the samples spiked with additional dhurrin (Fig. 3b).

### Time-resolved transcriptome analysis of developing grain

To investigate the expression profiles of genes encoding enzymes involved in dhurrin biosynthesis and putative turnover processes, transcriptome analysis of the developing grain was performed. More than 871 million 100 bp paired-end reads were generated resulting in an average of 58 million reads per library. Of these reads, an average of 87% were mapped to the Phytozome sorghum transcriptome v2.1, with 55% being uniquely mapped and 31% non-specifically mapped. Of the predicted 33,032 protein-coding gene loci in sorghum, 31,088 loci (94%) were detected as expressed during sorghum grain development. Among these transcripts, a large fraction showed an expression below a single fragment per kilobase of transcript per million mapped reads (FPKM) (9,283 reads, 28%). With these values considered as the background level, 11,127 genes (34%) were registered as not being expressed. The results obtained from the transcriptome study were verified by quantitative PCR (qPCR) using specific primers towards the gene CAS (*Sobic.006G016900*) (Additional file 1A).

**Fig. 4:**
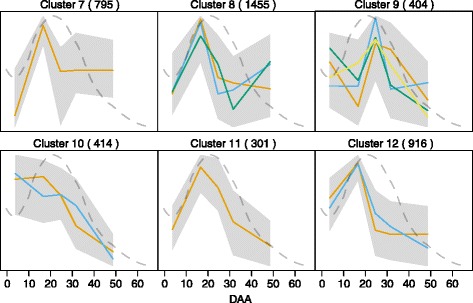
Clustering analysis of transcript expression profiles. The hierarchical clustering is divided into 20 groups, which was further subdivided into 81 subgroups (colored lines in each cluster). Only the cluster groups containing the biosynthetic genes (CYP79A1, CYP71E1 and UGT85B1) is displayed on the figure. The complete overview of all cluster groups can be found in Additional file 3. The number of genes in each cluster is listed in parenthesis next the cluster number. The *grey field* represents the span of all the expression profiles in a single cluster. The *dashed line* represents the concentration of dhurrin pr. mg tissue through the grain development in relation to the expression profiles

**Fig. 5:**
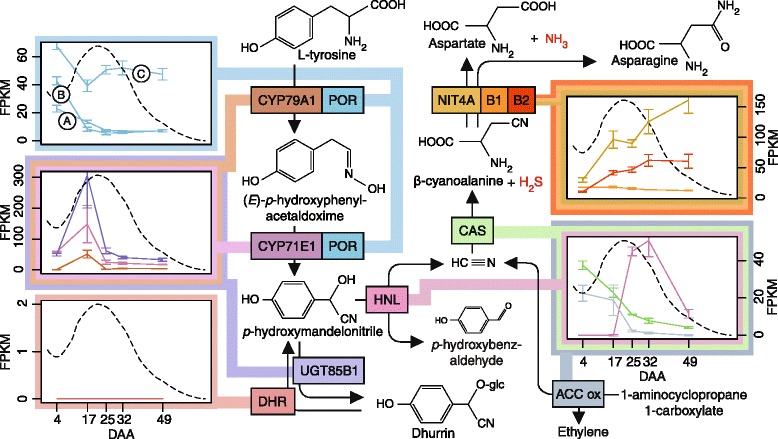
Expression profiles linked to the biosynthetic- and bioactivation pathway. The biosynthetic pathway involves the enzymes CYP79A1, CYP71E1 and UGT85B1 with the formation of dhurrin from L-tyrosine. The bioactivation pathway hydrolyzes dhurrin to release HCN via the enzymes DHR1 and 2 and HNL. The detoxification pathway converts the HCN to aspartate + NH3 and/or asparagine via CAS and the NIT4A/B1 and NIT4A/B2 heterodimers. The expression profiles for the specific enzyme involved in the reactions in the biosynthetic- or bioactivation pathway are colored in the same color as the enzyme boxes. In the *box* representing the POR expression, the **a**, **b** and **c** represents the three different isoforms found in sorghum. Expression is displayed in fragments per kilobase of exon per million reads mapped (FPKM). The *dashed line* in each transcription box represents the dhurrin concentration throughout development

Functional genomics and identification of interesting genes was initially guided by categorizing the expressed genes according to their expression pattern throughout grain development. All expression profiles were normalized (Z-score normalized FPKM values) and clustered using hierarchical clustering with Wards method [31] (Additional file 2). The expression patterns of the cluster groups containing the biosynthetic genes (cytochrome P450 (CYP) 79A1 (*Sobic.001G012300*) and UGT85B1 (*Sobic.001G012400*) in 8–1 and CYP71E1 (*Sobic.001G012200*) in 12–1) as well as nitrilase (NIT) 4B1 (*Sobic.004G225000*) in 10–1 is shown along with the concentration of dhurrin in Fig. 4. These six cluster groups shared an overall similarity (B-1 on Additional file 2). The remaining 14 cluster group profiles were contained in three larger groups (A-1, B2 and B3 Additional file 2) which are presented in Additional file 3. It is to be noted that the NADPH-dependent cytochrome P450 oxidoreductase (POR) isoforms crucial for the dhurrin biosynthesis were not contained in these clusters, but did exhibit maximum gene expression before the dhurrin concentration peak and belonged to the same overall group as the biosynthetic genes (B in Additional file 2). This difference in the expression profiles is more easily observed in Fig. 5.

In the bio-activation pathway, the two DHR genes known to be present in the sorghum genome were not expressed, while the HNL transcript reached maximum abundance a few days after the dhurrin concentration peak and then decreased in conjunction with dhurrin. In contrast, the two genes encoding the two subsequent enzymes CAS and NIT4B1 in the detoxification pathway showed maximum expression at anthesis with a steady decline towards grain maturation. A similar trend was seen for 1-aminocyclopropane-1-carboxylic acid (ACC) oxidase. This enzyme represents the final enzymatic step in ethylene biosynthesis and releases stoichiometric amounts of HCN, which is detoxified, via the same CAS detoxification pathway [32, 33]. The transcripts for NIT4A and NIT4B2 displayed a third type of expression profile with an almost linearly increased expression from anthesis to grain maturation (Fig. 5).

For many of the genes displayed, the standard deviations of their expression profiles were relatively low. The two genes CYP71E1 and UGT85B1 in the dhurrin biosynthesis were exceptions and displayed relatively large standard deviations at 17 DAA (Fig. 5). Despite these large standard deviations, the three genes in the dhurrin biosynthesis displayed a relatively fixed ratio between the transcript abundances from 17 DAA, with CYP79A1 being the lowest expressed and UGT85B1 the highest expressed gene, with CYP71E1 having an intermediate expression. From western blot analysis using available antibodies against CYP79A1 and CYP71E1, a similar distribution was found with CYP71E1 having the highest concentration (Additional file 4A-D). However, the protein ratio was on average 4-fold lower compared to the RNA ratio. In terms of maximum abundance, CYP71E1 and UGT85B1 displayed large values of several hundred FPKM as opposed to the POR isoforms, HNL, CAS, NIT4B1, NIT4B2 and ACC oxidase, which all displayed transcript abundances below 100 FPKM (Fig. 5).

### Glutathione S-transferases in *S. bicolor*

**Fig. 6:**
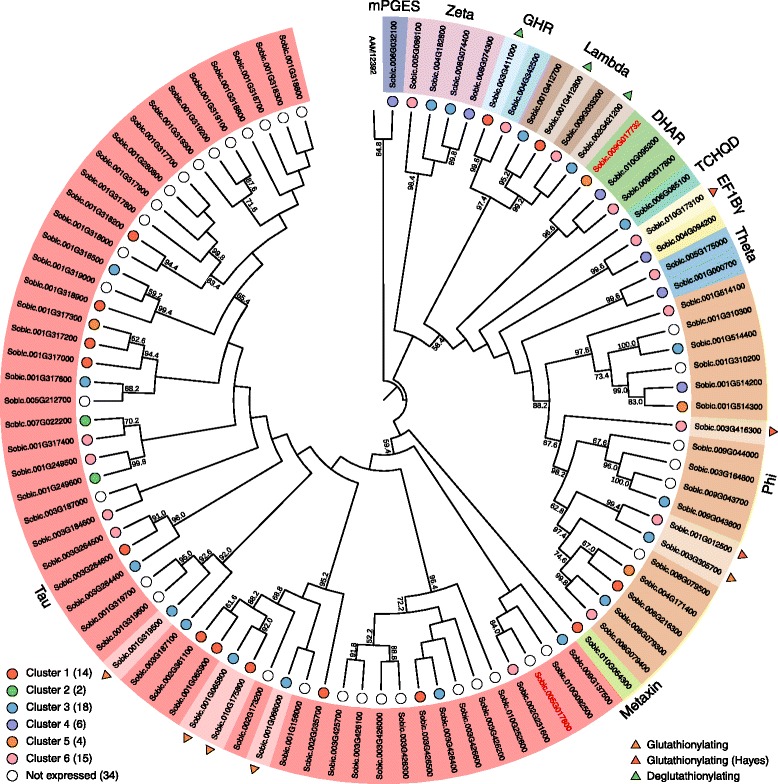
Phylogenetic analysis of Sorghum bicolor glutathione-S transferases. The phylogenetic tree represents the 93 GSTs found in sorghum. The tree is constructed with phyML using 500 bootstrapping replicates. The bootstrapping values are displayed in percentage and only values above 50 are displayed. The prokaryotic glutaredoxin (accession: AAM12392), was used as an outgroup to root the tree. The two gene names marked in *red* color represent one gene (*Sobic.005G017800*) where only the N-terminal GST domain was detected and a gene (*Sobic.009G017732*), with a new gene name in Phytozome database version 3.1. The *colored circles* represent the cluster groups displayed in Fig. 7. *Uncolored circles* represent genes not expressed in the grain during development. The *triangular shapes* on the outside of the phylogenetic tree represent the GST genes potentially involved in dhurrin turnover. Their specific functions are listed in the bottom right legend

A total of 110 genes encoding putative GSTs were identified in the *S. bicolor* genome by BlastP searches using a selection of GST protein sequences from *Arabidopsis thaliana* [34], barley [35]*,* rice (*Oryza sativa*) [36], black poplar (*Populus trichocarpa*) [37], *Physcomitrella patens* [38] and a selection of 215 other plant, animal, fungi, and bacteria species [38]. A Conserved Domain search at the National Center for Biotechnology Information (NCBI) database and a Pfam search at the European Bioinformatics Institute database revealed that 13 of the selected sorghum protein sequences did not contain any GST domains whereas four only contained the C-terminal domain. These genes were retraced to the new Phytozome genome v3.1, to test if they were annotated incorrectly in the old database. The retracing did not change the sequences and these enzymes are likely catalytically inactive and were therefore deleted from the collection. Two sorghum sequences only contained an N-terminal domain (*Sobic.005G017800*, *Sobic.009G017900*). Retracing these genes revealed that *Sobic.005G017800* is not annotated in the new database, while *Sobic.009G017900* is replaced by *Sobic.009G017732*. Domain search revealed that this gene has both N- and C-terminal domains. Both these genes were included in the phylogenetic- and transcript expression analyses. All 93 sorghum GST genes were classified into 36 distinct classes via an NCBI conserved domain search and a phylogenetic analysis using the list of 215 full-length GST described by Liu et al. [38] (Fig. 6, *Sobic.005G017800* and *Sobic.009G017732* marked by red font). The phylogenetic tree revealed that sorghum contained eleven GST classes (number of sequences in parenthesis): microsomal ProstaGlandin E-Synthase (mPGES) (1), Zeta (4), Glutathionyl-Hydroquinone Reductases (GHR) (2), Lambda (4), DeHydroAscorbate Reductases (DHAR) including *Sobic.009G017732* (3), TetraChloro-HydroQuinone Dehalogenases (TCHQD) (1), Elongation Factor 1B Gamma (EF1Bγ) (2), Theta (2), Phi (18), Metaxin (1) and Tau (55, including one only containing the N-terminal). In general, the positions of the individual genes in the outer branches of the tree have good support with high bootstrap values, but the relation between the different GST classes in the tree were not very well supported. Among the 93 GSTs, the Tau and Phi genes were by far the most numerous representing 77% of the total number of genes.

A previous genome-wide survey, identified 99 GSTs in sorghum [39]. Their division of the GSTs into the different classes was based on phylogenetic analysis of only the N-terminal parts of the proteins although the division was classically based on the entire sequences. As an example, they identified only two Lambda GSTs [34, 40], none of which were identical to the four identified in the present study. Furthermore, the sequences classified as Lambda GSTs by Chi et al. [39] have a serine residue in the active site rather than the catalytic cysteine characteristic of Lambda GSTs and were therefore incorrectly classified as a consequence of the N-terminal based phylogenetic analysis. In our analyses, these two genes were categorized as Tau GSTs. The validity of our approach was confirmed by identical numbers and gene ids of cys GSTs (DHAR, GHR, Lambda and mPGES) to those reported by Lallement et al. [40]. Our analysis also revealed a new GST class (Metaxin) not previously described in sorghum.

### Expression analysis of GSTs in *S. bicolor*

**Fig. 7:**
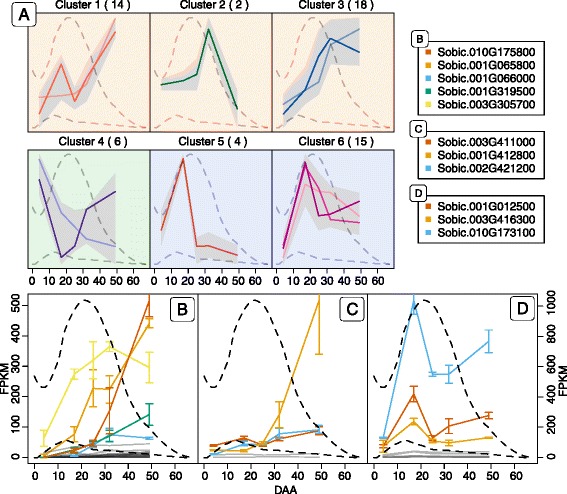
Expression profiles of GSTs in the course of sorghum grain development. All 59 expressed GSTs found in the phylogenetic analysis were normalized to Z-score and clustered using hierarchical clustering with Wards method (**a**). The hierarchical clustering was divided into 6 groups, which was further subdivided into 12 subgroups (*colored lines* in each cluster) (Additional file 6). The *lighter colored line* represents the first subgroup and the darker colored line the last subgroup in a cluster. The number of genes in each cluster is listed in parenthesis next to the cluster number. The *grey* field represents the span of all the expression profiles in a single cluster. The *dashed lines* represent the concentration of dhurrin (largest peak between day 10 and 20) and pGlcPAAc through grain development in relation to the expression profiles of the different GSTs. Glutathionylating GSTs from cluster 1–3 plotted in FPKM (**b**). Deglutathionylating GSTs from cluster 1–3 plotted in FPKM (**c**). GST identified by Hayes et al. (2015) along with similar genes in similar cluster group 6 (**d**). Highly expressed genes in panel **b**–**d** have colored lines referring to the genes listed next to panel **a**. *Gray lines* are genes only expressed at low abundance

Based on the transcriptome analyses of the sorghum grain from anthesis to maturation, the expression patterns of the 93 sorghum GSTs were assigned to different cluster groups. This was done to guide the identification of possible gene candidates for involvement in the putative turnover pathway. Of the 93 GST genes found, 34 were not expressed during the development of the grain. Group clustering of the remaining 59 genes (Fig. 7a and Additional file 5) into six groups revealed that the genes were primarily distributed among clusters 1, 3 and 6. From the dendrogram (Additional file 5), it was possible to determine the relatedness between the cluster groups. Cluster group 1–3 were related and all displayed expression profiles that have the highest abundance after the concentration of dhurrin peaked (orange background). In contrast, the two related cluster groups 5 and 6 displayed the highest abundance prior to the dhurrin peak (blue background), while cluster group 4 displayed an intermediate expression profile with high abundance both before and after the dhurrin peak (green background).

The GSTs found in cluster group 1–3 were of most interest as their expression followed that of NIT4A and NIT4B2 and increased over time, at least until after the dhurrin concentration had peaked. In GST enzymes that catalyze glutathionylation, a serine or tyrosine is the catalytic residue, whereas deglutathionylating GSTs harbor a catalytic cysteine [34, 40]. The classes Zeta, TCHQD, EF1Bγ, Theta, Phi, Metaxin and Tau belong to the first group, while the classes DHAR, Lambda, GHR and mPGES belong to the second group of GSTs. The expression profiles of glutathionylating and deglutathionylating GSTs from cluster 1–3 are shown in Fig. 7b and c, respectively, with the most abundant members displayed in colored lines. The five most abundant genes in Fig. 7b included four Tau class GSTs (*Sobic.010G175800*, *Sobic.001G065800*, *Sobic.001G319500*, *Sobic.001G066000*) and a Phi class GST (*Sobic.003G305700*). Among the GST genes from cluster 1–3, the ones containing a cysteine residue were depicted in Fig. 7c. Of these genes, the most abundant ones were displayed in colored lines. These genes included two Lambda class GSTs (*Sobic.001G412800* and *Sobic.002G421200*) and a GHR class GST (*Sobic.003G411000*). Hayes et al. [25] suggested that the Phi class GST gene *Sobic.001G012500*, lying close to the gene cluster containing the dhurrin biosynthetic genes (CYP79A1 - *Sobic.001G012300*, CYP71E1 - *Sobic.001G012200* and UGT85B1 - *Sobic.001G012400*) [41], could be involved in dhurrin turnover in the tissue. This gene was found in cluster group 6, a cluster group, which shared a similar initial expression profile to the biosynthetic genes (Fig. 5). The genes from cluster group 6 are plotted in Fig. 7d. The two most abundant genes beside the GST described by Hayes et al. [25] include a Phi class GST (*Sobic.003G416300*) and an EF1Bγ class GST (*Sobic.010G173100*). When the expression profiles of all 59 GST genes were compared to the average expression profile of NIT4A and NIT4B2 using Pearson correlation, the glutathionylating GSTs in Fig. 7b are ranked as the 7th (*Sobic.001G319500*), 9th (*Sobic.001G065800*), 12th (*Sobic.003G305700*), 13th (*Sobic.001G066000*) and 22nd (*Sobic.010G175800*) most similar (Additional file 6). In the deglutathionylating group in Fig. 7c, *Sobic.002G421200* was found to be the GST most similar to the two NIT expressions with *Sobic.003G411000* ranked as 16th and *Sobic.001G412800* as 25th. In comparison the three deglutathionylating GSTs in Fig. 7d (*Sobic.010G173100*, *Sobic.001G012500* and *Sobic.003G416300*) were only ranked as 35th, 38th and 47th, respectively (Additional file 6).

### Nitrilase activity in a novel turnover pathway

**Fig. 8:**
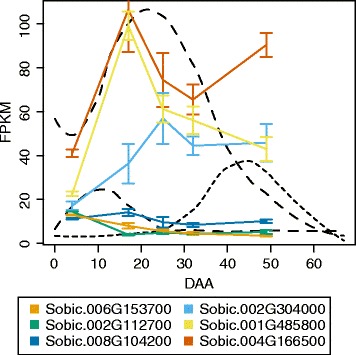
Expression profiles of the sorghum nitrilase superfamily genes in the course of grain development. The expression profiles of the six nitrilase superfamily genes expressed in the course of grain development shown in FPKM. The colors of the profiles corresponds to the gene names listed in the box below the plot. The three *dashed curves* represent dhurrin (*tallest dashed curve*), dhurrin amide (*left-most dashed curve*) and dhurrin amide (*right-most dashed curve*). The NIT4 genes are excluded from this figure, but shown in Figs. 5 and 11

The dhurrin turnover products, dhurrin amide and dhurrin acid (Fig. 2) are envisioned to be produced by the action of either a nitrilase or by the combined action of a nitrile hydratase and an amidase. To find putative nitrilase candidate genes in the sorghum genome, it was blasted using protein sequences ascribed to each of the 12 branches of the nitrilase superfamily, which include amidases and cyano hydratases but not nitrile hydratases [42]. To classify the ten genes discovered, they were aligned against the known nitrilase superfamily sequences and compared to the consensus sequences around the EKC catalytic triad [43] (Additional file 7). The ten genes were categorized as four branch-1 nitrilases (NIT4A, NITB1, NITB2 and *Sobic.006G153700*), a single branch-5 β-ureidoproprionase (*Sobic.002G304000*), a branch-8 glutamine-dependent NAD synthetase (*Sobic.002G112700*), three branch-10 Nit (branch defined by eukaryote protein with unknown function) (*Sobic.001G485800*, *Sobic.008G104200* and *Sobic.010G077400*) and a single branch-11 N-carbamyl putrescine amidohydrolase (*Sobic.004G166500*). Nine of the ten genes were expressed during grain development (*Sobic.010G077400* not expressed). Three of these nine genes belong to the well-characterized NIT4 family (NIT4A, NIT4B1 and NIT4B2) involved in the detoxification pathway and one of the two putative turnover pathways. These three genes were therefore not included (Fig. 8). The six genes were divided into three low abundance genes (*Sobic.006G153700*, *Sobic.002G112700* and *Sobic.008G104200*) and three high abundance genes (*Sobic.004G166500*, *Sobic.001G485800* and *Sobic.002G304000*).

### UDP-glycosyltransferase expression and possible involvement in metabolite formation

**Fig. 9:**
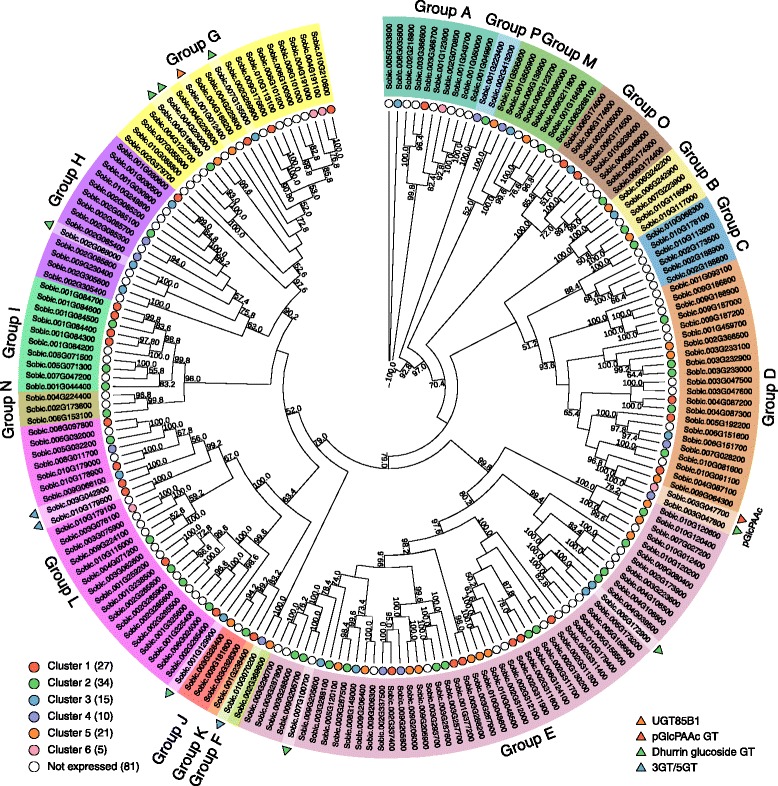
Phylogenetic analysis of Sorghum UDP-glycosyltransferases. The phylogenetic tree represents the 193 UGTs found in sorghum. The tree is constructed with phyML using 500 bootstrapping replicates. The bootstrapping values are displayed in percentage and only values above 50 are displayed. The phylogenetic tree is divided into separate groups of UGTs from A-M plus O. The *colored circles* represent the cluster groups displayed in Fig. 10. *Uncolored circles* represent genes not expressed in the grain. In the *bottom left legend*, the number of expressed genes in each cluster group is listed. The *triangular shapes* on the outside of the phylogenetic tree represent the UGT genes potentially involved in dhurrin and flavonoid metabolism. Their specific names are listed in the *bottom right legend*

The concentration of pGlcPAAc was observed to accumulate to maximum levels before the dhurrin concentration of the grain reached its maximum (Fig. 2). To identify potential UGT candidates capable of glucosylating pOHPAAc, a total of 205 genes encoding putative UGTs were identified in the sorghum genome by blast searches using 97 *A. thaliana* UGT protein sequences [44] and two sequences from *Malus domestica* and *Glycine max* representing the novel O group UGT [45] (Additional file 6). Alignment and domain analyses revealed that 12 sequences were not recognized as UGTs by the domain analyses. Additionally, two sequences were missing the first conserved residues of the PSPG motif located near the C-terminal which is involved in binding the nucleotide sugar donor [46]. These sequences were excluded from further analyses. The remaining 193 genes were subjected to phylogenetic analyses along with the previous reference sequences (Fig. 9) [45]. The relationship between the individual genes is generally supported by high bootstrap values, while the relationship between the different UGT classes has lower bootstrap support. All 16 UGT groups previously defined [45] were represented in the phylogenetic tree. Ten sequences were assigned to group A, 5 to group B, 6 to group C, 24 to group D, 50 to group E, 2 to group F, 20 to group G, 14 to group H, 10 to group I, 3 to group J, 1 to group K, 27 to group L, 8 to group M, 3 to group N, 8 to group O and 2 to group P (Fig. 9).

**Fig. 10:**
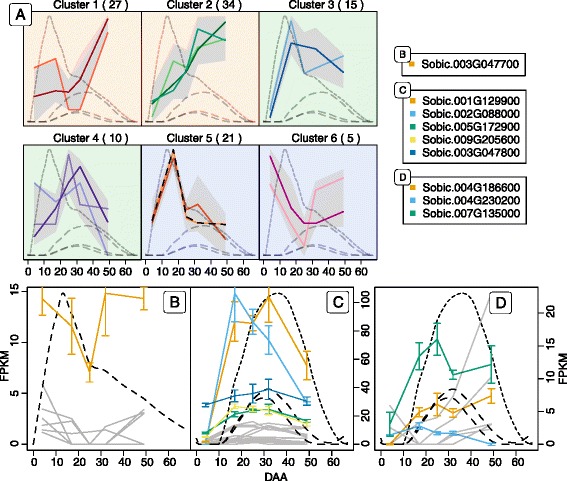
Expression profiles of sorghum UGTs. All 108 expressed UGTs found in the phylogenetic analysis were normalized to Z-score and clustered using hierarchical clustering with Wards method (**a**). The hierarchical clustering was divided into 6 groups, which was further subdivided into 14 subgroups (*colored lines* in each cluster) (Additional file 8). The *lighter color* represents the first subgroup and the *darker color* the last subgroup in a cluster. The number of genes in each cluster is listed in parenthesis next the cluster number. The *grey field* represents the span of all the expression profiles in a single cluster. The *dashed curves* represent the concentration of pGlcPAAc (largest peak between day 10 and 20) and the three dhurrin glucosides (smaller peaks) pr. mg tissue in the course of grain development in relation to the expression profiles. Genes in cluster 1 plotted in FPKM values of interest to the conversion of the glucosylation of pOHPAAc (**b**). Genes from cluster 3 plotted in FPKM values representing good candidates for the formation of the three CNdglcs (**c**). UGTs contained in phylogenetic group G (defined in Fig. 9) (**d**). The colors in **b**–**d** refers to the gene names listed next to panel **a**

To narrow down the number of possible gene candidates, the expression data from the transcriptome study were used to cluster the UGT genes according to their expression patterns (Fig. 10a). Of the 193 UGT genes found, 81 were not expressed in the grain in the course of development. The remaining 112 genes were clustered into six distinct cluster groups, which were further subdivided into 15 subgroups (Additional file 8). The expression profiles of cluster group 1 and 2 were related (Additional file 8) and both increased towards the end of grain development (Fig. 10a). In comparison, cluster group 3 and 4 had expression profiles, which peaked over a broader range from 20 to 40 DAA. Cluster group 5 and 6 peaked in the early development from 0 to 20 DAA.

Because pGlcPAAc reached maximum levels at the early phase of grain development, the gene encoding this UGT was expected to have a high initial expression. Cluster group 1–1, 4–1 and 6 meet these criteria (Fig. 10a). Only one (*Sobic.003G047700*) of the five genes from these cluster groups was expressed throughout development (Fig. 10b), albeit at a low abundance compared to the earlier described genes. This gene belonged to the Group D UGTs. The other four all had time points where they were not expressed and were only expressed in low abundance (Fig. 10b grey lines).

The dhurrin glucosides accumulated late in the course of grain development, when the dhurrin content was decreasing (Fig. 2). Genes encoding the potential UGT candidates capable of catalyzing glucosylation of dhurrin were therefore expected to display maximum expression later than the maximum expression time point for UGT85B1 (marked by black dashed lines in cluster 5 Fig. 10a). Genes matching this trend were found in cluster groups 3–2, 4–2 and 4–3 (Fig. 10a). Combined this selection contained 22 genes, of which five were expressed with a maximum abundance above 20 FPKM (*Sobic.001G129900*, *Sobic.002G088000*, *Sobic.005G172900, Sobic.009G205600*, *Sobic.003G047800*) (Fig. 10c) and belonged to Group L, H, E, E and D, respectively (Fig. 9). None of the genes expressed at high abundance from the previous two selections (Fig. 10b and c) belonged to the Group G UGTs. The genes in group G were of particular interest, because UGT85B1 involved in the biosynthesis of dhurrin resided in this class. As dhurrin is most likely the substrate for the formation of the dhurrin glucoside, it is possible that a UGT capable of glucosylation of dhurrin would share sequence similarities with UGT85B1. All genes from this group were therefore displayed together in Fig. 10d to reveal UGT gene candidates not discovered via the cluster analysis. Of particular interest were three gene candidates (*Sobic.004G186600, Sobic.004G230200, Sobic.007G135000*) expressed at high abundance and correlated with the concentration of the dhurrin glucoside during early development (Fig. 10d).

### Gene expression profiles of genes involved in the dhurrin catabolism

**Fig. 11:**
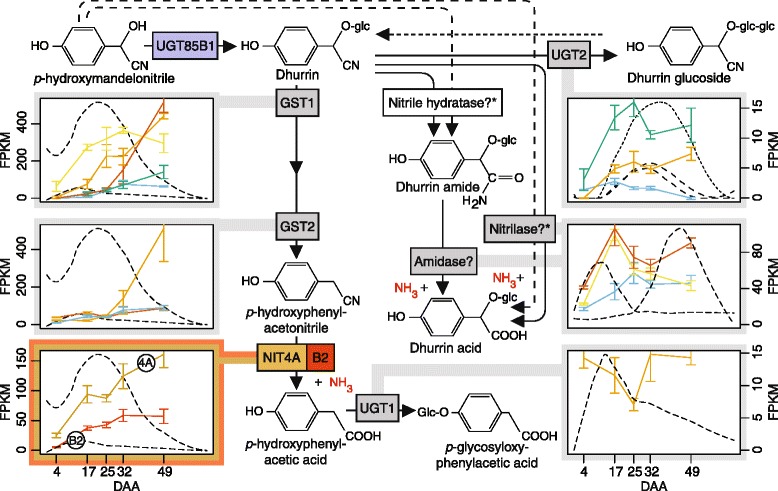
Metabolite accumulation and expression of genes in the two putative dhurrin turnover pathways. The transcript *boxes* are simplified versions of the earlier figures: GST1 (Fig. 7b), GST2 (Fig. 7c), NIT4A/B2 (Fig. 5), UGT1 (Fig. 10b), UGT2 (Fig. 10d) and nitrilase/amidase (Fig. 8). The dhurrin acid and amide can in principle be formed via two different routes: from dhurrin or directly from pOHMn, *which then requires an additional glycosylation step in order to get the dhurrin acid and amide

All the expression profiles of the putative gene candidates described in the previous sections have been incorporated with their possible positions within the putative turnover pathways hypothesized by Jenrich et al. [24] and Pičmanová et al. [19] showing the catabolism of dhurrin (Fig. 11). Only the most promising gene candidates involved in the catabolism are displayed in Fig. 11. Of the GST genes selected as potentially involved in conversion of dhurrin to pOHPCN, three of the glutathionylating ones and one deglutathionylating GST have expression values above 200 FPKM, while the two other GST genes and NIT4A and NIT4B2 have a maximum abundance above100 FPKM. In comparison, the UGT gene candidate and the putative nitrilase/amidase genes involved in the second turnover pathway display a lower maximum abundances below 100 FPKM.

### Transcriptome analysis of the genes involved in the flavonoid pathway

To investigate the transcriptional regulation of the biosynthesis genes involved in the relatively unknown flavonoid pathway in sorghum and to support the possible role of PAs as defense compounds in the grain (Fig. 1), the sorghum genome was blasted with a selection of protein sequences involved in the above metabolic pathways in different plant species. All the matching sorghum genes were aligned with species characteristic amino acid sequences and a phylogenetic analysis conducted for all genes found for each of the individual enzymatic steps (Additional file 9A-L). The sorghum genes most closely related to monocot sequences and in particular to maize were extracted from the analysis and their expression profiles plotted in the flavonoid biochemical pathway (Additional file 10).

The gene encoding the initial enzyme chalcone synthase (CHS) in the flavonoid metabolism had previously been identified in sorghum [47, 48]. This enzyme converts the two substrates *p*-coumaroyl-CoA and malonyl-CoA into naringenin-chalcone. Eight isoforms of the enzyme have previously been identified, but several other gene candidates exist in the genome (Additional file 9A). Only two of these identified genes (SbCHS5, *Sobic.005G136200* - purple and SbCHS6, *Sobic.005G137300* - blue) were expressed in the grain in high transcript abundance and both peaked before the second concentration peak in PAs between 17 and 25 DAA. Five sequences were found for the second enzyme, chalcone isomerase (CHI) which converts naringenin-chalcone to naringenin (Additional file 9B). One of these genes had been identified earlier (*Sobic.001G035600* - red) [47]. Another transcript (*Sobic.008G030100* - black) was expressed in equally high abundance to the identified gene, but this gene resided in the clade with dicotyledons and is a less likely candidate. The B-phenyl ring of naringenin can be hydroxylated at the 3’ or at the 3’- and 5’ positions to produce eriodictyol or dihydrotricetin, respectively. These hydroxylations are performed by flavonoid 3’-hydroxylase (F3’H) and flavonoid 3’,5’-hydroxylase (F3’5’H). In sorghum, four putative F3’H and 16 F3’5’H (Additional file 9C) were identified. Of the four F3’H genes, 3 had previously been identified in sorghum (SbF3’H1 - *Sobic.004G201100* - purple, SbF3’H2 - *Sobic.004G200800* - green and SbF3’H3 - *Sobic.004G200900* - blue) [49]. None of the 16 F3’5’H genes had previously been described, but *Sobic.007G029900* (orange) and *Sobic.001G360700* (pink) were closely related to maize and barley F3’5’H sequences. Two of the F3’H genes (*Sobic.004G201100* and *Sobic.004G200800*) might be of importance to the hydroxylation of the flavonoids destined for PAs as they peaked in correlation with the PAs. For the F3’5’H genes, only the gene *Sobic.001G363700* (red) displayed an expression profile of interest, but this was not closely related to any of the known monocot F3’5’H sequences (Additional file 9C). The naringenin, eriodictyol and dihydrotricetin followed the same basic pathway and ended up as the three different catechins, which upon conjugation form the PAs. In the following description of the steps leading up to the formation of the catechins, only the one originating from naringenin is mentioned as the enzymes involved were similar.

From naringenin, it is possible to form three different compounds. Flavone synthase II (FNSII) converts naringenin to the flavone apigenin. The gene *Sobic.002G000400* (SbFNSII – green, Additional file 9D) had previously been identified [50] and displayed an increasing abundance of transcript from 20 DAA towards maturation. The concentration of flavones has not previously been measured in the grain, but the transcript level co-occurred with the appearance of the grain coat pigmentation, which started around 17 DAA and increased towards maturation (Fig. 1c). A second gene (*Sobic.006G001000* – blue, Additional file 9D) could be of interest because of its relatedness to the maize FNSII gene *GRMZM2G407650* and shared a similar expression profile to the characterized sorghum FNSII gene. The final transcript (Sobic.001G363700 - red), resided in the same monocotyledon clade, and although it was not very closely related to other known transcripts, it shared an expression profile correlated with that of CHS and CHI (Additional file 9D).

Naringenin is further converted to dihydrokaempferol by flavanone 3-hydroxylase (F3H). Two genes *Sobic.006G253900* (SbF3H1 - red) and *Sobic.006G254000* (SbF3H2 - blue) encoding F3H had earlier been identified in sorghum [47] and in the current study displayed profiles correlated with the PAs (Additional file 9E). Dihydrokaempferol can be converted to flavonols by flavonol synthases (FLS). Two genes were found, but only one was expressed and at a very low abundance (*Sobic.004G310100* – blue, Additional file 9F). Following the pathway of PAs, dihydrokaempferol is converted to leucopelargonidin by dihydroflavonol reductase (DFR). Of the four genes encoding DFR previously identified in sorghum (SbDFR1 and SbDFR3 had been characterized), three were expressed, *Sobic.003G230900* (SbDFR1 - red), *Sobic.003G231000* (SbDFR2 - green) and *Sobic.004G050200* (SbDFR3 - blue) (Additional file 9G) [47]. Only the two transcripts DFR1 and DFR2 displayed expression profiles correlated with the concentration of PAs.

The leucopelargonidin produced is further converted to pelargonidin by anthocyanidin synthase (ANS). The ANS encoding gene (*Sobic.004G000700*, SbANS - red) had previously been identified in sorghum [47] and is the best candidate due to a high initial abundance. Six other putative ANS sequences were also found, but they only shared an average amino acid similarity to SbANS of 33 ± 1% and were positioned in a separate clade in the monocot clade (Additional file 9H). Pelargonidin can be glucosylated by flavonoid-3-*O*-glucosyltransferase (3GT) and flavonoid-5-*O*-glucosyltransferase (5GT) to produce anthocyanins. No 3- or 5GT genes had previously been identified in sorghum, but eight putative genes were found in our analysis, with six being expressed in the grain (Additional file 9I). The two genes *Sobic.010G070200* (purple) and *Sobic.002G369600* were positioned close to maize and barley 3GT, in the clade containing all other 3GT genes and belonged to Group F UGTs (Fig. 9). Only *Sobic.010G070200* was expressed in the grain and displayed an expression profile that correlated with the pigment formation in the grain coat. Two of the other genes (*Sobic.003G042900* - orange and *Sobic.010G179500* - pink) were likely 5GTs as they group together with other 5GT genes (Additional file 9I). They both belong to Group L UGTs (Fig. 9) and had an increase in transcript abundance towards the end of grain maturation.

For the formation of PAs, pelargonidin is further converted to (−)-epiafzelechin by anthocyanidin reductase (ANR). Eleven ANR genes were discovered (Additional file 9J), with eight expressed and four of these having a relevant transcript expression (*Sobic.006G227200* - blue, *Sobic.006G227300* - purple, *Sobic.006G227400* - green, *Sobic.006G227100* - red). The epicatechin produced from pelargonidin by ANR have a cis orientation of the B-phenyl ring. A second enzyme, the leucoanthocyanidin reductase (LAR) is capable of producing catechins, which have a trans orientation of the B-phenyl ring. LAR enzymes are found in barley and rice [51], but our phylogenetic analysis did not identify any such genes in sorghum (Additional file 9K).

The last enzymatic step resulting in the formation of PAs has still not been elucidated, but laccases (LACs) have previously been determined to play a role in polymerization of PAs in several plant species [52, 53]. In sorghum, 27 LAC encoding genes were found of which 19 were not expressed in the grain (Additional file 9L). Of the remaining eight genes, two (*Sobic.001G403100* - red and *Sobic.004G236000* - lime) had high transcript abundances, but only *Sobic.001G403100* displayed a profile correlated with the PA concentration.

## Discussion

### Dhurrin biosynthesis and accumulation in the developing grain

The content of CNglcs within a plant species varies ontogenetically, phenologically, and chronologically. Although many exceptions can be found, the concentration of CNglcs is in general highest in seedlings and decreases with plant age [1]. CNglcs may accumulate in any part of a plant and newly formed tissues are typically more cyanogenic compared to older tissues [1, 20, 21, 54–56].

Forage and grain sorghum plants are known to be cyanogenic due to their content of the CNglc dhurrin [57, 58]. The dhurrin content of the mature sorghum grain has been shown to be negligible or zero [4, 28, 29]. In the current study, it was discovered that dhurrin accumulate in the immature grain (Fig. 1). The dhurrin content of the husk, which is the maternal tissue component of the grain, was close to zero throughout the entire grain development and thus not correlated with the amount of dhurrin in the grain. From the transcriptome analysis, it is also evident that the genes encoding the enzymes involved in dhurrin biosynthesis in vegetative tissue (CYP79A1, CYP79E1, POR and UGT85B1) were expressed in high abundances immediately before the concentration of dhurrin peaks (Fig. 5). This substantiated that dhurrin is *de novo* biosynthesized in the grain from tyrosine via the pathway discovered in etiolated seedlings of sorghum [59, 60].

Similar to sorghum, the mature seeds of *Lotus japonicus*, cassava (*M. esculenta*) and barley (*H. vulgare)* contain no or only negligible amounts of CNglcs [55, 61, 62]. In bitter almonds, the cyanogenic monoglucoside prunasin is biosynthesized in the tegument of the almond fruit throughout development to reach a maximum HCNp of 16 nmol/mg [7]. At the end of maturation, prunasin is transported to the developing cotyledons of the kernel where it is converted and stored as amygdalin, which reaches a maximum HCNp of 89 nmol/mg [7]. In mature seeds of rubber tree (*H. brasiliensis*), the cotyledon is similarly high in CNglcs with a HCNp of 132 nmol/mg tissue [63]. From these examples, it is clear that the sorghum grain only contains low amounts of CNglcs.

Earlier research into the biosynthesis of dhurrin concluded that the biosynthesis of dhurrin in the vegetative tissue is largely controlled at the transcriptional level [21]. This earlier finding is confirmed by our results, where large standard deviations in the transcript levels of CYP71E1 and UGT85B1 coincided with the large deviations in the dhurrin concentration at day 17 (Figs. 1 and 5). Furthermore, these large standard deviations in the gene transcript abundance was only seen at day 17 and was therefore not attributable to technical variation or a general biological variation observed for every time point during grain development. Despite these variations, the ratio between the transcript abundances of CYP79A1, CYP71E1 and UGT85B1 were relatively fixed and showed an incremental increase of transcript from the first (CYP79A1) to the last enzyme in the biosynthesis (UGT85B1) (Fig. 5). The same tendency was seen in the ratio between proteins (Additional file 5: Figure S5) and could be a safety feature to ensure that none of the toxic intermediates (*E*-*p*-hydroxyphenylacetaldoxime and *p*-hydroxymandelonitrile (pOHMn)) from CYP79A1 and CYP71E1 are released during biosynthesis, but converted by the downstream processes to dhurrin.

### Sorghum grain is not cyanogenic

Cyanogenesis is the classical defense mechanism assigned to CNglcs and is based on their hydrolysis by BGD and HNL resulting in the release of HCN and ketones [10]. The specific BGDs hydrolyzing dhurrin are assigned as DHRs in sorghum [10]. The absence of DHR activity in the developing grain was demonstrated by the observed lack of hydrogen cyanide release from grain macerates (Fig. 3). Furthermore, the lack of an expected quantitative HCN release upon addition of almond BGD must reflect the presence of endogenous inhibitors in the grain such as the high amounts of PAs (Fig. 1) [64, 65]. The missing hydrolysis of dhurrin in grain homogenates was also substantiated by the complete absence of transcripts encoding DHR. Although transcripts encoding the enzymes HNL, CAS and NIT4B1 were all found to be expressed in the grain, their expression profiles indicate that they are not directly involved in dhurrin turnover in the grain (Fig. 5). Instead, the expression profile of HNL in relation to the dhurrin concentration curve and the lack of DHRs indicate that it might serve as a scavenger of pOHMn, released by chemical degradation of dhurrin during turnover. The expression profiles of CAS and NIT4B1 correlate with ACC oxidase and indicate that these enzymes are primarily involved in detoxification of HCN from ethylene production and not cyanogenesis.

In other cyanogenic plants, the endogenous BGDs are extremely active and will rapidly hydrolyze any freely available CNglcs. In the past, this prevented the use of CNglcs as the key substrates in experiments with such extracts aimed at detecting the possible operation of endogenous turnover pathways. The lack of cyanogenesis in the developing sorghum grain renders this a unique experimental system to study CNglc synthesis as well as endogenous turnover.

### Involvement of two alternative pathways in the turnover of dhurrin

The observed disappearance of dhurrin in the course of grain maturation, the absence of BGD activity and the lack of correlation between CAS and NIT4B1 expression with the dhurrin disappearance must reflect the operation of one or more dhurrin turnover pathways that do not proceed via HCN release. The operation of such pathways has previously been proposed [1, 19, 21, 24]. Interestingly, the expression profiles of the genes encoding the NIT4A/NIT4B2 heterodimer indicate that they are involved in a continuous turnover of dhurrin throughout grain development and not only when the concentration of dhurrin decreases at 25 DAA (Fig. 11). This is supported by the finding that the glucosylated NIT4A/NIT4B2 product, pGlcPAAc, accumulates in the early stages of grain development while the dhurrin content is still increasing (Fig. 2). A similar result was presented by Adewusi [20], who showed that dhurrin turnover proceeded in young plants while the total amount of dhurrin was still increasing. Although the transcript abundances indicate that the nitrilases might be involved in dhurrin turnover, no traces of pOHPCN and pOHPAAc were found in the grains indicating that they might be transient intermediates. However, the applied LC-MS method does not effectively detect the free pOHPAAc. It is therefore possible that in the developing grain, this precursor of pGlcPAAc is either accumulated in its free form instead of being glucosylated or incorporated into other compounds.

With the lack of DHR activity and transcript in the sorghum grain, the hypothesis that conversion of dhurrin to the nitrilase substrate pOHPCN should be mediated by this enzyme in concert with e.g. a protein co-factor must be rejected. We instead propose the involvement of GSTs as supported by Hayes et al. [25] and have identified a number of candidate GST genes. The transcriptome data suggested the involvement of both glutathionylating and deglutathionylating GSTs in this metabolic transformation. The expression profile of one highly expressed deglutathionylating Lambda GST (*Sobic.002G421200*) was ranked as the GST most correlated with the expression of NIT4A and NIT4B2, and *Sobic.001G319500* and *Sobic.001G065800* highly expressed glutathionylating GSTs were likewise strongly correlated (7th and 9th). These high similarities to the expression patterns of NIT4A and NIT4B2 are consistent with their possible involvement in continuous turnover of dhurrin. The Phi class GST (*Sobic.001G012500*) and the other GSTs in cluster group 6 (Fig. 7d), did not show a favorable expression pattern and had a low similarity to the NIT expression profiles and are therefore not as obvious gene candidates for the glutathionylation as the GST mentioned above. Future expression of the best gene candidates and characterization of the enzymes will reveal if they are indeed involved in dhurrin turnover.

In vegetative tissues, pGlcPAAc accumulates as the sorghum plants get older and dhurrin is turned over [19, 24, 26]. The time restricted production of this compound in grain, guided identification of potential UGT candidates for the glucosylation of pOHPAAc into pGlcPAAc. Our transcriptome analyses demonstrated that 108 UGT encoding genes were expressed in the developing grain (Fig. 9). In comparison, 60 of the 137 genes encoding UGTs in flax (*Linum usitatissimum*) are expressed in the developing seeds [66]. From the phylogenetic- and hierarchical clustering analyses only a single UGT (group D) gene candidate (*Sobic.003G047700*) was found with an expression profile correlated with the concentration of pGlcPAAc (Fig. 10b). In *A. thaliana*, group D UGTs is known to glucosylate a range of different aglycones including terpenoids, flavonoids, benzoates and brassinosteroids and it is therefore possible that this sorghum UGT is responsible for production of pGlcPAAc.

Besides the pathway just discussed, a second putative turnover pathway involving the dhurrin amide and dhurrin acid probably exists [19]. The formation of these two dhurrin derivatives may be envisioned from two possible precursors, either dhurrin or directly from pOHMn (Fig. 11). In both scenarios the nitrile-function can be converted into a carboxylic acid group by the direct action of a nitrilase or via the amide by the combined action of a nitrile hydratase and an amidase [67]. Interestingly, a nitrile hydratase capable of producing an amide from a nitrile has never been characterized in any plant species, although such enzymatic activities have been measured in several species including sorghum [68]. Only the cyano hydratases, which are both structurally and mechanistically different from the nitrile hydratases, have been characterized in plants [23, 24]. In bacteria, the formation of indole-3-acetamide from indole-3-acetonitrile occurs via the action of a nitrile hydratase [69]. A BlastP search of the sorghum proteome using known bacterial nitrile hydratase protein sequences gave no significant hits (results not shown). The bacterial nitrile hydratases are metallo-containing enzymes and thus both structurally and mechanistically different from the nitrilase superfamily enzymes from plants, e.g. NIT4A, NIT4B1 and NIT4B2. Enzymes in this family are the best candidates for the production of the amide and acid derivatives [42, 43]. Only one new gene candidate was found in this family (*Sobic.006G153700*). The abundance of this gene makes it unlikely, as a candidate for the formation of dhurrin acid, but it could however be involved in the formation of the dhurrin amide. Similarly, the glutamine-dependent NAD synthetase (*Sobic.002G112700*) could be involved in the dhurrin amide formation as this type of enzyme hydrolyze amides to produce the corresponding acid and ammonia [42]. The last two low abundance nitrilases (*Sobic.008G104200*) belong to the branch 10 nitrilases with unknown functions and has the potential to be involved. For the gene *Sobic.002G112700* protein homologs with high sequence similarity (≈95%) were found in acyanogenic monocots, whereas the sequence similarity for the genes *Sobic.008G104200 and Sobic.006G153700* were lower (≈82% and ≈ 77% respectively) (Source: Phytozome). The last two genes are therefore better gene candidates for the formation of the dhurrin amide, but needs to be cloned and expressed before any conclusions can be drawn. All the high abundance genes had a maximum abundance peaking after the dhurrin amide and are therefore probably not involved in its formation, but could still be involved in conversion of this compound into dhurrin acid. However, none of them showed expression profiles correlated with the accumulation of dhurrin acid and protein homologs with high similarities were found in other acyanogenic plants (*Sobic.001G485800* ≈ 84%; *Sobic.004G166500* ≈ 94%; *Sobic.002G304000* ≈ 95%) (Source: Phytozome). As *Sobic.001G485800* has the lowest sequence similarity, this would be the first gene to clone and express for activity.

In conclusion, only dhurrin acid accumulated with a profile directly matching the degradation of its presumed precursor dhurrin. This was in contrast to the previous results in studies using vegetative sorghum tissues where the level of pGlcPAAc continued to increase as dhurrin was turned over [19, 24, 26]. The content of dhurrin acid was not analyzed by Jenrich et al. [24], but in the two other cases, the content of this compound also increased with age. Our current metabolite analyses indicated that in developing sorghum grain, the turnover pathway leading to pGlcPAAc formation was dominating during dhurrin accumulation, whereas the pathway producing dhurrin acid was dominating at the later stages.

### A dual function for dhurrin in the developing grain

Although the HCNp of the developing sorghum grain is low compared to seeds and fruits of other cyanogenic plants, they still contain a potentially lethal content of dhurrin. Reports indicate that 1–3 mg of cyanide per kilogram body mass is lethal to most vertebrates [1]. This means that only 15–45 grains each containing 1 μg dhurrin could be lethal to a small 15 g bird. This may be the reason that the African bird *Quelea quelea* avoids feeding on developing grains of sorghum [70]. The lack of sorghum BGDs can be compensated for by presence of BGDs in the saliva and gut of the birds and other herbivores, which would activate the toxicity of dhurrin. This remains a hypothesis, as we were not able to demonstrate a stoichiometric HCN release from dhurrin upon addition of almond BGD to sorghum grain homogenates indicating the presence of inhibitors, which may or may not inhibit the release upon consumption by birds. The deterrent effect of dhurrin may be augmented by the presence of high amounts of PAs, which have previously been shown to deter birds and other grain predators [71]. Our analysis did not quantify the PA concentration, as no standards were available, but research show that sorghum grains have a very high PA abundance compared to other food plants [72]. In other cyanogenic species, CNglcs and PAs may function in a similar trade-off strategy, where they complement or even compensate each other [73, 74]. The Chaparral shrub, *Heteromeles arbutifolia* is one such example [75]. In the immature fruit, the pulp accumulates high amounts of PAs and CNglcs. Once the fruits mature and turn red, a rapid turnover of PAs occur in the pulp and the CNglcs are transported to the seed. The fruit is thus safe for birds to eat and the seed will be dispersed and remain protected by CNglcs until germination. In sorghum, it is tempting to speculate that the combined effects of dhurrin and PAs protect the more attractive immature sorghum grain while the PAs alone are sufficient to protect the mature grain against herbivores.

Similar to the dhurrin biosynthetic gene transcripts, transcripts involved in the flavonoid pathway peaked prior to the concentration maximum of the PAs at 20 DAA (Additional file 10). Few reports are available on the expression of all the flavonoid genes in course of plant ontogeny and of these, the majority only compare the differential expression between tissue types and treatments [76–78]. In the flowers of cotton (*Gossypium spp.*), expression of the genes in the flavonoid pathway responsible for the production of anthocyanins correlated with the concentration of anthocyanin, while ANR and FLS1 showed different expression profiles [79]. For sorghum, the genes CHS, CHI, F3’H, FNSII, F3H, DFR, ANS have previously been described in other publications, but none of them have been characterized. The research presented here add additional gene candidates to the aforementioned range of enzymes, but also pinpoints some genes which are likely to the primary ones involved in the flavonoid biosynthesis in the developing grain. Furthermore, novel gene candidates including the 3GT/5GT, ANR and LAC were also identified which were previously unknown in sorghum and could help the understanding of the PA biosynthesis.

The dramatic turnover of dhurrin in the developing sorghum grain is initiated at the time point where the PA content peaked. At this developmental stage the PAs are envisioned to protect the sorghum grain towards herbivory. The function of dhurrin as a defense compound may then be shifted towards a function as a nitrogen resource. This dual function could be a cost-effective measure during the energy demanding grain-filling period, saving the plant from simultaneous production of both defensive compounds and storage proteins [80]. Furthermore, the PAs do not contain nitrogen and can thus be biosynthesized without the need for substrate containing nitrogen, which is needed for storage protein production. The accumulation of dhurrin means that nitrogen is still being assimilated in the grain, albeit in a form unavailable to herbivores. As the grain matures and becomes less attractive due to lower water content and higher concentrations of PAs, the nitrogen in dhurrin can be remobilized as ammonia and utilized to produce storage proteins. In the developing sorghum grain, storage protein in the form of protein bodies first occurs in the endosperm 15 DAA and continue to increase in concentration towards maturation [81]. Assimilation of nitrogen from the soil in cereals is often insufficient to supply the developing grain and the majority of the nitrogen in the grain originates from leaf tissue remobilization [82]. It is therefore possible that the accumulation and remobilization of dhurrin in the early developing sorghum grain supplies it with a more direct and readily available source of nitrogen for the formation of protein bodies in the later stages.

To supply endosperm cells with nitrogen, the dhurrin glucosides could function as a transport form of dhurrin from the site of biosynthesis at the ER membranes to the vacuoles for storage [83]. For the glucosylation of dhurrin, eight potential UGT gene candidates were discovered (Fig. 10c and d). Although the expression profiles of two of the genes (*Sobic.004G186600* and *Sobic.004G230200*) correlated with the concentration of dhurrin diglucosides, only *in-vitro* assays can determine the precise substrate specificity of these enzymes. As three different structural configurations of the CNdglcs exist, it is possible that three different UGTs also have to be found among the eight gene candidates. In most plants, the formations of the different glycosides are catalyzed by distinct UGTs. This is the case for the production of anthocyanins in the flavonoid pathway, where the 5-*O* and 3-*O* glycosylations are performed by two different enzymes (Additional file 10) [84]. To remobilize the nitrogen in dhurrin, the dhurrin glucosides are likely hydrolyzed and metabolized via the turnover pathways described (Fig. 11). The results obtained in this study suggest that the pathway producing pOHPAAc continuously metabolize dhurrin, to potentially act as a nitrogen buffer and supply the growing grain with reduced nitrogen. The metabolite accumulation in the secondary pathway suggests that the dhurrin amide and acid might have different substrate sources. The late accumulation of dhurrin acid indicates that this compound most likely is a turnover product from the degradation of dhurrin. On the contrary, the early accumulation of the dhurrin amide indicates that this might originate directly from pOHMn as a result of improper metabolon formation and could function as an additional nitrogen source. When the production of protein bodies starts 15 DAA, all available nitrogen sources are remobilized, including the dhurrin amide. As pGlcPAAc and dhurrin acid have already released their nitrogen, the turnover of these compounds may not be as urgent as the glucose moieties they contain are not a limiting source. They are therefore degraded at a slower rate and only decreases to zero at the time of maturation (Fig. 2).

## Conclusion

We have used the developing grains of *Sorghum bicolor* (L.) Moench to study the metabolism of dhurrin. Metabolite profiling using LC-MS/MS revealed that dhurrin is accumulated in high amounts during the early phases of grain development but is absent in the mature grain. Despite the high amount of dhurrin in the early developmental phase, the grains were acyanogenic and only released minute amounts hydrogen cyanide upon tissue maceration. This result was confirmed by the complete absence of transcripts encoding the dhurrin specific β-glucosidases (dhurrinases). Although the accumulated dhurrin may still have a secondary protective role and be toxic to potential grain predators via herbivore β-glucosidases, the primary function of dhurrin is probably as a nitrogen store. The loss of the protective functions of dhurrin could be replaced by the high amounts of proanthocyanidins accumulated in the grain via the flavonoid pathway. Several new gene candidates for this pathway were discovered in this study, including 3GT/5GT, ANR and LAC. With the missing activity of the dhurrinases, the turnover of dhurrin occurs via two alternative turnover pathways for which intermediates were confirmed by metabolite profiling. For the turnover of dhurrin via dhurrin amide and dhurrin acid, several novel gene candidates encoding enzymes from the nitrilase super family were discovered. Although their expression profiles are not ideal in relation to metabolite accumulation, their possible modes of action still prompt for further investigation. On the contrary, cluster analyses revealed strong candidates for the glucosylation of dhurrin to dhurrin glucoside, glucosylation of pOHPAAc acid and potential GSTs for sequential conversion of dhurrin to pOHPCN. By combining all the acquired results, we can conclude that one turnover pathway is likely to be involved in continuous turnover of dhurrin, while the other is primarily involved in turnover at later stages of grain development. The combined effects of these two putative turnover pathways could supply the developing grain with nitrogen to fuel the production of storage protein.

## Methods

### Plant material

Grains of the *S. bicolor* grain cultivar MR43 were sown February 2^nd^, 2011 in fields at Gatton College (latitude:-27.5412, longitude: 152.3355, Elevation, 94 m) in South East Queensland, Australia. Before sowing, the fields were supplied with the following amounts of fertilizer: 120 kg/ha of urea (46% nitrogen) and 120 kg/ha of CK 88 (15.1% N; 4.4% P; 11.5% K and 13.6% S). The panicles were collected in the period starting April 1^st^ before the flowers had opened to June 7^th^ when the grain had fully matured. Samples were collected 0, 0.5, 3, 4, 10, 13, 17, 20, 25, 32, 40, 45, 49, 52, 56, 61, 63 and 67 DAA. The panicles were on average 30 cm in length. After being harvested, each panicle was divided into five sections and immediately frozen in liquid nitrogen and stored at −80 °C. Only the top section of the panicle was used for the experiments in this study. For analysis of dhurrin content, the husk was removed from the grain under liquid nitrogen and analyzed separately. At each harvest time point, the physical appearance of the grains and husks was documented by photographing (Fig. 1).

### LC-MS/MS analysis of dhurrin related metabolites and colorimetric analysis of flavonoids in grains

For extraction of metabolites, grains harvested at time points 3–67 DAA were divided into smaller pieces under liquid nitrogen using a mortar and pestle. Husks and grains harvested at time points 0–0.5 DAA were extracted intact. At each time point, five grains and five husks were collected from each of three different panicle tops. All samples were extracted in 85% MeOH + 0.5% HCOOH. Material from husks and grains harvested 32–67 DAA were extracted trice in 500 μL solvent. Smaller grains were extracted trice in the following volumes: day 0: 50 μL; day 0.5: 100 μL; day 3: 150 μL, day 4: 200 μL; day 10: 250 μL; day 13: 300 μL; day 17: 350 μL; day 20: 400 μL and day 25: 450 μL. The biological material was extracted by adding the listed amount of cold solvent to an Eppendorf tube, weighing the closed tube, adding the frozen plant material and immediately subjecting it to boiling for 3.5 min in the closed Eppendorf tube. The tubes were cooled on ice and weighed to determine the fresh weight of the biological material. The MeOH was then extracted and the process repeated two additional times. The three extracts were combined, diluted five times with H_2_O and filtered (0.22 μm low-binding Durapore membrane) before analysis.

Analytical LC-MS/MS were performed using similar programs, equipment and detected via specific adduct ions as previously described Pičmanová et al. [19], except for dhurrin acid which was identified via an additional ion (375 [M + 2Na-H]^+^). The dhurrin calibration curve used for absolute quantification covered the range 0.8–400 μM (diluted 5 times for analysis). Local polynomial regression fitting using the “loess” function (https://stat.ethz.ch/R-manual/R-devel/library/stats/html/loess.html) in the R statistical programming software v. 3.1.1 (https://www.r-project.org) were used to calculate the trend line for the different compounds.

The content of PAs in developing grains and husks were quantified using colorimetry [85]. To account for the presence of anthocyanidins, anthocyanins and other compounds, which absorb light at 550 nm, the spectrophotometer was zeroed before each measurement using a blank reaction containing sample and reagents but not heated to 95 °C.

### Hydrogen cyanide assay

Three independent samples of 15 mg grain material collected at harvest time points 4, 13, 25, 40, 56 and 67 DAA were suspended in 200 μL MES buffer (pH 6.5) in 1.5 mL Eppendorf tubes (screw cap top lids) and incubated for 2.5 h at 30 °C with vigorous shaking (1,000 rpm). One set of samples contained only buffer, the second set with addition of 0.67 U/mL almond BGD (Sigma G8625, https://www.sigmaaldrich.com) and the third set spiked with an additional 5 nmol dhurrin and BGD. After incubation, the tubes were flash frozen in liquid nitrogen, 40 μL 6 M NaOH were added and the samples thawed at room temperature. The Eppendorf tubes were then centrifuged (2 min, 20,000 × g) and 60 μL aliquots transferred to the wells of a 96-well microtiter plate alongside aliquots of a KCN standard solution (0–18 nmol). Colorimetric determination of HCN was carried out as previously reported [86]. For the time point 25 DAA, the assay was repeated with 5 mg biological material, 5 U/mL BGD and a reduced incubation time of 0.5 h. To remove protein aggregates of grain proteins and BGD formed after the addition of HOAc (only formed with 10× higher concentration of BGD), the assays were performed in Eppendorf tubes and centrifuged (20,000 × g, 2 min) before absorbance measurements.

### Transcriptomic analysis

#### RNA extraction

Total RNA was extracted from triplicate samples of developing grains (4, 17, 25, 32 and 49 DAA) using the Spectrum™ Plant Total RNA Kit (Sigma) and incorporating a DNase digestion step with DNase I (Qiagen, https://www.qiagen.com). To extract RNA from the starch rich grains at time points beyond 4 DAA, a phenol extraction step was incorporated into the protocol. Plant material was solubilized in the provided kit buffer and homogenized with 500 μL Acid phenol (24:25:1, Ambion, http://www.thermofisher.com). The mixture was centrifuged (5 min, 20,000 × g, 4 °C) and the supernatant obtained mixed with 1 mL chloroform:isoamylalkohol (24:1 Fluka, http://www.sigmaaldrich.com) and centrifuged (2 min, 20,000 × g, 4 °C). The RNA containing supernatant was used for the subsequent steps in the protocol. The RNA extracts were stored at −80 °C. The yield and purity of each RNA sample were determined by the absorbance at 260 and 280 nm using a Nanodrop spectrophotometer (www.nanodrop.com). RNA integrity was quantified using a BioAnalyzer (Agilent, http://www.agilent.com) and the Agilent RNA 6000 Nano Assay. Only samples with a RNA integrity number (RIN) value above seven were used.

#### cDNA library preparation and sequencing

Isolated RNA was shipped on dry ice to Macrogen Inc. (Seoul, Korea) for cDNA library preparation and sequencing steps. The cDNA libraries were prepared according to the manufacturer’s instructions (Illumina, https://www.illumina.com). Briefly, poly(A) containing mRNA molecules were purified from each sample of total RNA (1–10 μg varying between samples). The mRNA was fragmented before cDNA synthesis primed by random primers. The resulting cDNA was modified for subsequent adapter-ligation using Illumina paired end adapters. These were then size-selected using agarose gel electrophoresis before being enriched with 15 rounds of PCR amplification. Each library had an insert size ranging from 300 to 500 bp and 100 bp sequences were generated following the Illumina HiSeqTM 2000 platform sequencing protocols.

#### Data analysis

If not otherwise stated, the analytical tools were run using standard settings. All genomes, transcripts and annotations were acquired from Phytozome (http://phytozome.jgi.doe.gov). The raw sequence reads were trimmed for adapter sequences with the program AdapterRemoval (version 1.1) using the settings --minlength 30, −-minquality 20, −-trimns, −-mm 3 [87]. The output sequences were further processed using PRINSEQ-lite (version 0.20.4) with the options -trim_left 10, −trim_qual_right 20, −min_len 30 [88]. The filtered sequence reads were mapped to the Phytozome sorghum transcriptome v2.1 [89] using Bowtie 2 [90]. Bowtie 2 was run with a maximum insert size between 300 and 500 bp (according to the specific library size). The transcript abundances of the alignments were quantified using the RNA-Seq quantification tools eXpress (version 1.5.1) [91]. eXpress was run using the bowtie 2 alignments, the Phytozome sorghum transcriptome and a mean fragment length according to the library size. The generated raw gene counts were normalized using the Bioconductor package edgeR [92] with the function calcNormFactors and transformed into FPKM with the edgeR package. FPKM values below 1 were considered as not expressed and assigned the value 0. FPKM counts for alternative transcripts were compiled to represent the counts for the single loci. The mean and standard deviation of the three replicates were used for further analyses.

### Quantitative PCR

The results from the RNA-sequencing experiment were confirmed using qPCR with primers specific for CAS gene (*Sobic.006G016900*) with normalization to the two reference genes ZF (*Sobic.007G187200*) and AAPK (*Sobic.003G200600*). New RNA was extracted following the same procedure as above. The concentration and purity of the RNA were analyzed using a Nanodrop spectrophotometer, while the integrity was analyzed on a 1% agarose gel following electrophoresis in TAE buffer at 60 V for 1 h and visualization by UV trans-illumination. cDNA was generated using the Bio-Rad iScript cDNA Synthesis Kit (http://www.bio-rad.com). Before synthesis, all RNA samples were diluted to a concentration of 500 ng/μL. Each reaction contained 2 μg RNA, 4 μL 5× iScript Reaction Mix, 1 μL iScript Reverse Transcriptase and nuclease free water (total volume: 20 μL). The samples were run on a Bio-Rad Tetrad 2 Peltier thermal cycler for 5 min at 25 °C, 30 min at 42 °C and 5 min at 85 °C. For the qPCR experiment, the following primers were used: CAS 5’-CATTGTGACTGTTCTTCCAAGCCT-3’ and 5’-GTTAGTTTGGCCCGTGACCTT-3’; ZF 5’- TCCACCAGCACTCAGGTTTC-3’ and 5’- GTTTTCATGGCTCAGGTCGA-3’; AAPK 5’- GGAATGGTGCATTCATGCCG-3’ and 5’- ACGCACCATTGGTAATCCTCC-3’. A Rotor-Gene Q (Qiagen) equipped with a Rotor-disc 72 was used for the qPCR. The primer specificity was determined by melting curve analyses, running the qPCR product of each primer on a 4% agarose gel and Sanger sequencing of the qPCR product (performed by Macrogen Inc.). A standard template mixture of 1 μL cDNA from each of the 15 extractions was used for the analysis. Each qPCR reaction contained: 10 μL SYBR® Green Master Mix (Qiagen), 7 μL H_2_O, 1 μL template (50 ng/μL) and 1 μL Fwd primer (10 μM) and 1 μL Rev primer (10 μM). The samples were run at: 95 °C for 7 min, 40 cycles of 95 °C for 10 s, 60 °C for 15 s, 72 °C for 30 s (fluorescence measured on green channel) followed by 60 °C for 1 min and melting curve from 60 to 95 °C. The qPCR product was purified with the QIAquick® PCR Purification Kit (Qiagen), analyzed on a 4% agarose gel, and sequenced by Sanger sequencing (Macrogen Inc.). The efficiency of each primer was determined by qPCR using serial dilutions. The PCR product was first diluted 100× in H_2_O with 10 μg/ml sheared salmon DNA (Ambion AM9680) and followed by dilutions in H_2_O to 1:10^3^, 1:10^4^, 1:10^5^, 1:10^6^, 1:10^7^. The dilutions were then used for qPCR using the procedure above. Each dilution was run in triplicate and the efficiency determined with the formula: Efficiency = −1 + 10^(−1/slope).^ For the actual qPCR experiment, all samples for each gene were analyzed in a single run with each time sample run in triplicate, a RT(−) run in duplicate and a no template control (NTC) run in duplicate. Only two biological replicates were used for the time point 49 DAA, to have room for all samples in a single run. Quantification of gene expression was achieved using the equation described by Pfaffl [93].

### Phylogenetic and expression analyses

Sorghum gene candidates putatively involved in dhurrin endogenous turnover and flavonoid biosynthesis were identified by BlastP (version 2.2.28) searches against the sorghum proteome v2.1 [89] using amino acid sequences and E-cutoff value > 1e-50. Information on the enzyme types involved in the sorghum flavonoid pathway was extracted from the KEGG PATHWAY Database (http://www.genome.jp/kegg-bin/show_pathway?sbi00941). The sequences were aligned using T-COFFEE (version_11.00.8cbe486) [94] in accurate mode for GST and NIT sequences and mcoffee mode for the remaining sequences. GST sequence alignments were further adjusted manually using BioEdit (v. 7.2.5) [95]. The substitution model of amino acid substitution was selected with ProtTest (v. 3.4) [96]. Phylogenetic trees were constructed using maximum likelihood method with PhyML (v. 3.1 20150325) [97] and 500 bootstrap replicates to obtain confidence support. The bootstrap values for individual trees were transformed to percentage for easier viewing. The prokaryotic glutaredoxin (accession: AAM12392) was used as an outgroup in the phylogenetic analyses of the GSTs [98]. Individual trees were rearranged into either monocotyledon or dicotyledon clades or into clades representing different enzyme classes, i.e. the F3’H and F3’5’H enzymes. Clustering of expression data was done using hierarchical clustering with the function hclust (https://stat.ethz.ch/R-manual/R-devel/library/stats/html/hclust.html) in R statistical programming software (v. 3.1.1).

### Total protein extraction and Western blotting

Total protein was extracted from six developing grains at harvesting time points 13, 25 and 49 DAA using phenol extraction [99] with an extraction buffer containing: 0.7 M sucrose, 5% (w/v) SDS, 0.1 M Tris–HCl (pH 8.0), 50 mM EDTA, 20 mM DTT and 1 mM PMSF. Before extraction, the grains were ground in liquid nitrogen with 20 mg PVPP. The protein pellet resulting from the phenol extraction was solubilized in 8 M urea, 4% (w/v) CHAPS, 20 mM DTT. Total protein concentration was quantified using a Bicinchoninic acid assay (Sigma Aldrich). An aliquot from each sample, corresponding to 200 μg of protein at 25 DAA was mixed with XT sample buffer (Bio-Rad), incubated at room temperature for 1 h and electrophoresed on a precast Criterion XT 12% gel (Bio-Rad) in MOPS buffer for 1 h at 200 V. Western blots were obtained as previously described [100] using antibodies towards CYP79A1 and CYP71E1 in 1:2000 dilution.

## Additional files

Additional file 1: A-F). Expression profile of CAS measured by qPCR, RNA integrity, concentration and purity, melting curve analysis, qPCR product run on 4% agarose gel, Sanger sequencing results of qPCR products and efficiency test of CAS, ZF and AAPK primers. (PDF 528 kb)
Additional file 2: Dendrogram showing specific clusters from the hierarchical clustering of all genes expressed in sorghum. (PDF 429 kb)
Additional file 3: Clustering analysis of transcript expression profiles divided into 20 groups of similar expression. (PDF 245 kb)
Additional file 4: A-C. Transcriptional and proteomic ratio between CYP71E1 and CYP79A1. The protein ratio is calculated from integrated areas of Western blot analysis of CYP79A1 and CYP71E1 using imageJ. (PDF 758 kb)
Additional file 5: Dendrogram showing specific clusters from the hierarchical clustering of expressed GSTs in sorghum. (PDF 150 kb)
Additional file 6: Overview of the sorghum GSTs and UGTs found in our analysis, with class specification, domain information and full-length sequence. Also included are Genebank annotations and full-length sequences used for the different analyses. (XLSX 551 kb)
Additional file 7: Consensus sequences around the EKC catalytic triad of the nitrilase superfamily defined by genes found in sorghum. (PDF 231 kb)
Additional file 8: Dendrogram showing specific clusters from the hierarchical clustering of expressed UGTs in sorghum. (PDF 153 kb)
Additional file 9: A-L. Phylogenetic trees for the genes involved in flavonoid metabolism. (PDF 347 kb)
Additional file 10: Expression profiles for genes involved in the flavonoid biosynthesis in developing sorghum grain. (PDF 657 kb)

## Abbreviations

3GT: Flavonoid-3-O-glucosyltransferase
5GT: Flavonoid-5-O-glucosyltransferase
ACC: 1-aminocyclopropane-1-carboxylic acid
ANR: Anthocyanidin reductase
ANS: Anthocyanidin synthase
BGD: β-glucosidase
CAS: β-cyanoalanine synthase
CHI: Chalcone isomerase
CHS: Chalcone synthase
CNdglc: Cyanogenic diglycoside
CNglc: Cyanogenic glycoside
CYP: Cytochrome P450
DAA: Days after anthesis
DFR: Dihydroflavonol reductase
DHAR: Dehydroascorbate reductase
DHR: Dhurrinase
EF1Bγ: Elongation Factor 1B Gamma
F3’,5’H: Flavonoid 3’,5’-hydroxylase
F3’H: Flavonoid 3’-hydroxylase
F3H: Flavanone 3-hydroxylase
FLS: Flavonol synthase
FNSII: Flavone synthase II
FPKM: Fragment per kilobase of transcript per million mapped reads
GHR: Glutathionyl-hydroquinone reductase
GO: Gene ontology
GST: Glutathione S-transferase
HCN: Hydrogen cyanide
HCNp: Cyanide potential
HNL: α-hydroxynitrile lyase
LAC: Laccase
LAR: Leucoanthocyanidin reductase
mPGES: Microsomal prostaglandin e-synthase
NCBI: National Center for Biotechnology Information
NIT: Nitrilase
PA: Proanthocyanidin
pOHMn: *p*-hydroxymandelonitrile
pOHPAAc: *p*-hydroxyphenylacetic acid
pOHPCN: *p*-hydroxyphenylacetonitrile
POR: NADPH-dependent cytochrome P450 oxidoreductase
qPCR: Quantitative PCR
TCHQD: Tetrachloro-hydroquinone dehalogenase
UGT: UDP-glycosyltransferase

## References

[1] Gleadow RM, Møller BL (2014). Cyanogenic glycosides: synthesis, physiology, and phenotypic plasticity. Annu Rev Plant Biol.

[2] Jones DA (1998). Why are so many food plants cyanogenic?. Phytochemistry.

[3] Nielsen KA, Olsen CE, Pontoppidan K, Møller BL (2002). Leucine-derived cyano glucosides in barley. Plant Physiol.

[4] Erb N, Zinsmeister HD, Nahrstedt A (1981). The cyanogenic glycosides of Triticum, Secale and Sorghum. Planta Med.

[5] Kojima M, Poulton JE, Thayer SS, Conn EE (1979). Tissue distributions of dhurrin and of enzymes involved in its metabolism in leaves of Sorghum bicolor. Plant Physiol.

[6] Jørgensen K, Morant AV, Morant M, Jensen NB, Olsen CE, Kannangara R (2011). Biosynthesis of the cyanogenic glucosides linamarin and lotaustralin in cassava: isolation, biochemical characterization, and expression pattern of CYP71E7, the oxime-metabolizing cytochrome P450 enzyme. Plant Physiol.

[7] Sanchez-Perez R, Jørgensen K, Olsen CE, Dicenta F, Møller BL (2008). Bitterness in almonds. Plant Physiol.

[8] Nahrstedt A (1970). Cyanogenesis in Prunus avium. Phytochemistry.

[9] Dziewanowska K, Niedzwiedz I, Lewak S (1979). Hydrogen cyanide and cyanogenic compounds in seeds. III. Degradation of cyanogenic glucosides during apple seed stratification. Physiologie Vegetale.

[10] Morant AV, Jørgensen K, Jørgensen C, Paquette SM, Sanchez-Perez R, Møller BL (2008). β-Glucosidases as detonators of plant chemical defense. Phytochemistry.

[11] Leavesley HB, Li L, Prabhakaran K, Borowitz JL, Isom GE (2008). Interaction of cyanide and nitric oxide with cytochrome c oxidase: implications for acute cyanide toxicity. Toxicol Sci.

[12] Tattersall DB, Bak S, Jones PR, Olsen CE, Nielsen JK, Hansen ML (2001). Resistance to an herbivore through engineered cyanogenic glucoside synthesis. Science.

[13] Zagrobelny M, Bak S, Rasmussen AV, Jørgensen B, Naumann CM, Møller BL (2004). Cyanogenic glucosides and plant-insect interactions. Phytochemistry.

[14] Møller BL (2010). Functional diversifications of cyanogenic glucosides. Curr Opin Plant Biol.

[15] Selmar D, Lieberei R, Biehl B (1988). Mobilization and utilization of cyanogenic glycosides - the Linustatin pathway. Plant Physiol.

[16] Selmar D (1993). Transport of cyanogenic glucosides: linustatin uptake by Hevea cotyledons. Planta.

[17] Swain E, Poulton JE (1994). Utilization of amygdalin during seedling development of prunus serotina. Plant Physiol.

[18] Selmar D, Irandoost Z, Wray V (1996). Dhurrin-6'-glucoside, a cyanogenic diglucoside from Sorghum bicolor. Phytochemistry.

[19] Pičmanová M, Neilson EH, Motawia MS, Olsen CE, Agerbirk N, Gray CJ et al. A recycling pathway for cyanogenic glycosides evidenced by the comparative metabolic profiling in three cyanogenic plant species. Biochem J. 2015;469(3):375-89. 10.1042/BJ20150390 26205491

[20] Adewusi SRA (1990). Turnover of dhurrin in green sorghum seedlings. Plant Physiol.

[21] Busk PK, Møller BL (2002). Dhurrin synthesis in sorghum is regulated at the transcriptional level and induced by nitrogen fertilization in older plants. Plant Physiol.

[22] Castric PA, Conn EE, Farnden KJF (1972). Cyanide metabolism in higher plants: V. The formation of asparagine from β-cyanoalanine. Arch Biochem Biophys.

[23] Piotrowski M, Schonfelder S, Weiler EW (2001). The Arabidopsis thaliana isogene NIT4 and its orthologs in tobacco encode β-Cyano-L-alanine hydratase/nitrilase. Journal of Biological Chemistry.

[24] Jenrich R, Trompetter I, Bak S, Olsen CE, Møller BL, Piotrowski M (2007). Evolution of heteromeric nitrilase complexes in Poaceae with new functions in nitrile metabolism. Proc Natl Acad Sci U S A.

[25] Hayes CM, Burow GB, Brown PJ, Thurber C, Xin Z, Burke JJ. Natural variation in synthesis and catabolism genes influences Dhurrin content in Sorghum. The Plant Genome. 2015;8(2) https://dl.sciencesocieties.org/publications/tpg/tocs/8/2. 10.3835/plantgenome2014.09.0048 33228310

[26] Blomstedt CK, O’Donnell NH, Bjarnholt N, Neale AD, Hamill JD, Møller BL et al. Metabolic consequences of knocking out UGT85B1, the gene encoding the glucosyltransferase required for synthesis of dhurrin in Sorghum bicolor (L. Moench). Plant and Cell Physiology*.* 2016. 10.1093/pcp/pcv153 26493517

[27] Patrick JW, Offler CE (2001). Compartmentation of transport and transfer events in developing seeds. J Exp Bot.

[28] Glennie CW (1983). Polyphenol changes in sorghum grain during malting. J Agric Food Chem.

[29] Panasiuk O, Bills DD (1984). Cyanide content of sorghum sprouts. J Food Sci.

[30] War AR, Paulraj MG, Ahmad T, Buhroo AA, Hussain B, Ignacimuthu S (2012). Mechanisms of plant defense against insect herbivores. Plant Signal Behav.

[31] Ward JH (1963). Hierarchical grouping to optimize an objective function. J Am Stat Assoc.

[32] Iqbal N, Trivellini A, Masood A, Ferrante A, Khan NA (2013). Current understanding on ethylene signaling in plants: the influence of nutrient availability. Plant Physiol Biochem.

[33] Matilla AJ (2000). Ethylene in seed formation and germination. Seed Sci Res.

[34] Dixon DP, Edwards R. Glutathione transferases. Arabidopsis Book. 2010;8. 10.1199/tab.0131 PMC3244946 22303257

[35] Rezaei MK, Shobbar ZS, Shahbazi M, Abedini R, Zare S (2013). Glutathione S-transferase (GST) family in barley: identification of members, enzyme activity, and gene expression pattern. J Plant Physiol.

[36] Soranzo N, Gorla MS, Mizzi L, De Toma G, Frova C (2004). Organisation and structural evolution of the rice glutathione S-transferase gene family. Mol Genet Genomics.

[37] Lan T, Yang ZL, Yang X, Liu YJ, Wang XR, Zeng QY (2009). Extensive functional diversification of the populus glutathione S-transferase supergene family. Plant Cell.

[38] Liu YJ, Han XM, Ren LL, Yang HL, Zeng QY (2013). Functional divergence of the glutathione S-transferase supergene family in physcomitrella patens reveals complex patterns of large gene family evolution in land plants. Plant Physiol.

[39] Chi Y, Cheng Y, Vanitha J, Kumar N, Ramamoorthy R, Ramachandran S (2011). Expansion mechanisms and functional divergence of the glutathione s-transferase family in Sorghum and other higher plants. DNA Res.

[40] Lallement PA, Brouwer B, Keech O, Hecker A, Rouhier N. The still mysterious roles of cysteine-containing glutathione transferases in plants. Front Pharmacol. 2014;5. 10.3389/fphar.2014.00192 PMC4138524 25191271

[41] Takos AM, Knudsen C, Lai D, Kannangara R, Mikkelsen L, Motawia MS (2011). Genomic clustering of cyanogenic glucoside biosynthetic genes aids their identification in Lotus japonicus and suggests the repeated evolution of this chemical defence pathway. Plant J.

[42] Brenner C (2002). Catalysis in the nitrilase superfamily. Curr Opin Struct Biol.

[43] Pace HC, Brenner C (2001). The nitrilase superfamily: classification, structure and function. Genome Biol.

[44] Ross J, Li Y, Lim EK, Bowles DJ (2001). Higher plant glycosyltransferases. Genome Biol.

[45] Caputi L, Malnoy M, Goremykin V, Nikiforova S, Martens S (2012). A genome-wide phylogenetic reconstruction of family 1 UDP-glycosyltransferases revealed the expansion of the family during the adaptation of plants to life on land. Plant J.

[46] Li Y, Baldauf S, Lim EK, Bowles DJ (2001). Phylogenetic analysis of the UDP-glycosyltransferase multigene family of Arabidopsis thaliana. Journal of Biological Chemistry.

[47] Liu H, Du Y, Chu H, Shih CH, Wong YW, Wang M (2010). Molecular dissection of the pathogen-inducible 3-deoxyanthocyanidin biosynthesis pathway in Sorghum. Plant and Cell Physiology.

[48] Lo C, Coolbaugh RC, Nicholson RL (2002). Molecular characterization and in silico expression analysis of a chalcone synthase gene family in Sorghum bicolor. Physiological and Molecular Plant Pathology.

[49] Shih C-H, Chu IK, Yip WK, Lo C (2006). Differential expression of two flavonoid 3 '-hydroxylase cDNAs involved in biosynthesis of anthocyanin pigments and 3-deoxyanthocyanidin phytoalexins in sorghum. Plant and Cell Physiology.

[50] Du YG, Chu H, Wang MF, Chu IK, Lo C (2010). Identification of flavone phytoalexins and a pathogen-inducible flavone synthase II gene (SbFNSII) in sorghum. J Exp Bot.

[51] Bogs J, Downey MO, Harvey JS, Ashton AR, Tanner GJ, Robinson SP (2005). Proanthocyanidin synthesis and expression of genes encoding leucoanthocyanidin reductase and anthocyanidin reductase in developing grape berries and grapevine leaves. Plant Physiol.

[52] Turlapati PV, Kim K-W, Davin LB, Lewis NG (2011). The laccase multigene family in Arabidopsis thaliana: towards addressing the mystery of their gene function(s). Planta.

[53] Hu Q, Luo C, Zhang Q, Luo Z (2013). Isolation and characterization of a Laccase gene potentially involved in proanthocyanidin polymerization in oriental persimmon (Diospyros kaki Thunb.) fruit. Mol Biol Rep.

[54] Jørgensen K, Bak S, Busk PK, Sørensen C, Olsen CE, Puonti-Kaerlas J (2005). Cassava plants with a depleted cyanogenic glucoside content in leaves and tubers. Distribution of cyanogenic glucosides, their site of synthesis and transport, and blockage of the biosynthesis by RNA interference technology. Plant Physiol.

[55] Forslund K, Morant M, Jørgensen B, Olsen CE, Asamizu E, Sato S (2004). Biosynthesis of the nitrile glucosides rhodiocyanoside A and D and the cyanogenic glucosides lotaustralin and linamarin in Lotus japonicus. Plant Physiol.

[56] Nahrstedt A (1985). Cyanogenic compounds as protecting agents for organisms. Plant Systematics and Evolution.

[57] Koukol J, Miljanich P, Conn EE (1962). The metabolism of aromatic compounds in higher plants: VI. Studies on the biosynthesis of dhurrin, the cyanogenic glucoside of Sorghum vulgare. Journal of Biological Chemistry.

[58] Haskins FA, Gorz HJ (1986). Relationship between contents of leucoanthocyanidin and dhurrin in Sorghum leaves. Theor Appl Genet.

[59] Møller BL, Conn EE (1980). The biosynthesis of cyanogenic glucosides in higher plants. N-Hydroxytyrosine as an intermediate in the biosynthesis of dhurrin by Sorghum bicolor (Linn) Moench. Journal of Biological Chemistry.

[60] Møller BL, Conn EE (1979). The biosynthesis of cyanogenic glucosides in higher plants. N-Hydroxytyrosine as an intermediate in the biosynthesis of dhurrin by Sorghum bicolor (Linn) Moench. Journal of Biological Chemistry.

[61] Forslund K, Jonsson L (1997). Cyanogenic glycosides and their metabolic enzymes in barley, in relation to nitrogen levels. Physiol Plant.

[62] Nartey F (1968). Studies on cassava, Manihot utilissima Pohl—I. Cyanogenesis: the biosynthesis of linamarin and lotaustralin in etiolated seedlings. Phytochemistry.

[63] Lieberei R, Selmar D, Biehl B (1985). Metabolization of cyanogenic glucosides in hevea brasiliensis. Plant Systematics and Evolution.

[64] Hargrove JL, Greenspan P, Hartle DK, Dowd C (2011). Inhibition of aromatase and alpha-amylase by flavonoids and proanthocyanidins from Sorghum bicolor bran extracts. J Med Food.

[65] Moini H, Guo QO, Packer L (2000). Enzyme inhibition and protein-binding action of the procyanidin-rich French maritime pine bark extract, pycnogenol: effect on xanthine oxidase. J Agric Food Chem.

[66] Barvkar VT, Pardeshi VC, Kale SM, Kadoo NY, Gupta VS. Phylogenomic analysis of UDP glycosyltransferase 1 multigene family in Linum usitatissimum identified genes with varied expression patterns. BMC Genomics. 2012;13. 10.1186/1471-2164-13-175 PMC3412749 22568875

[67] Kobayashi M, Shimizu S (1994). Versatile nitrilases: nitrile-hydrolysing enzymes. Fems Microbiology Letters.

[68] Agerbirk N, Warwick SI, Hansen PR, Olsen CE (2008). Sinapis phylogeny and evolution of glucosinolates and specific nitrile degrading enzymes. Phytochemistry.

[69] Kobayashi M, Suzuki T, Fujita T, Masuda M, Shimizu S (1995). Occurrence of enzymes involved in biosynthesis of indole-3-acetic acid from indole-3-acetonitrile in plant-associated bacteria, Agrobacterium and Rhizobium. Proc Natl Acad Sci U S A.

[70] Tarimo TMC, Cheke RA, RaMEK LJ (2000). The Cyanogenic Glycoside Dhurrin as a possible cause of Bird-resistance in Ark-3048 Sorghum. Research Priorities for Migrant Pests of Agriculture in Southern Africa.

[71] Bullard RW, York JO, Kilburn SR (1981). Polyphenolic changes in ripening bird-resistant Sorghums. J Agric Food Chem.

[72] Gu LW, Kelm MA, Hammerstone JF, Beecher G, Holden J, Haytowitz D (2004). Concentrations of proanthocyanidins in common foods and estimations of normal consumption. Journal of Nutrition.

[73] Briggs MA (1990). Chemical defense production in Lotus corniculatus L. I. The effects of nitrogen source on growth, reproduction and defense. Oecologia.

[74] Goodger JQD, Gleadow RM, Woodrow IE (2006). Growth cost and ontogenetic expression patterns of defence in cyanogenic Eucalyptus spp. Trees-Structure and Function.

[75] Dement WA, Mooney HA (1974). Seasonal variation in the production of tannins and cyanogenic glucosides in the chaparral shrub, Heteromeles arbutifolia. Oecologia.

[76] Abeynayake SW, Panter S, Chapman R, Webster T, Rochfort S, Mouradov A (2012). Biosynthesis of proanthocyanidins in white clover flowers: cross talk within the flavonoid pathway. Plant Physiol.

[77] Morohashi K, Casas MI, Falcone Ferreyra L, Mejia-Guerra MK, Pourcel L, Yilmaz A (2012). A genome-wide regulatory framework identifies maize pericarp color1 controlled genes. Plant Cell.

[78] Shi SG, Yang M, Zhang M, Wang P, Kang YX, Liu JJ. Genome-wide transcriptome analysis of genes involved in flavonoid biosynthesis between red and white strains of Magnolia sprengeri pamp. BMC Genomics. 2014;15. 10.1186/1471-2164-15-706 PMC4156625 25150046

[79] Tan J, Wang M, Tu L, Nie Y, Lin Y, Zhang X. The Flavonoid Pathway Regulates the Petal Colors of Cotton Flower. PLoS One*.* 2013;8(8). 10.1371/journal.pone.0072364 PMC3741151 23951318

[80] Neilson EH, Goodger JQD, Woodrow IE, Møller BL (2013). Plant chemical defense: at what cost?. Trends Plant Sci.

[81] Shull JM, Chandrashekar A, Kirleis AW, Ejeta G (1990). Development of Sorghum (Sorghum bicolor (L.) Moench) endosperm in varieties of varying hardness. Food Struct.

[82] Hawkesford MJ (2014). Reducing the reliance on nitrogen fertilizer for wheat production. J Cereal Sci.

[83] Saunders JA, Conn EE (1978). Presence of cyanogenic glucoside dhurrin in isolated vacuoles from Sorghum. Plant Physiol.

[84] Fukuchi-Mizutani M, Okuhara H, Fukui Y, Nakao M, Katsumoto Y, Yonekura-Sakakibara K (2003). Biochemical and molecular characterization of a novel UDP-glucose : anthocyanin 3 '-O-glucosyltransferase, a key enzyme for blue anthocyanin biosynthesis, from gentian. Plant Physiol.

[85] Porter LJ, Hrstich LN, Chan BG (1986). The conversion of procyanidins and prodelphinidins to cyanidin and delphinidin. Phytochemistry.

[86] Halkier BA, Møller BL (1989). Biosynthesis of the cyanogenic glucoside dhurrin in seedlings of Sorghum bicolor (L.) Moench and partial-purification of the enzyme-system involved. Plant Physiol.

[87] Lindgreen S (2012). AdapterRemoval: easy cleaning of next-generation sequencing reads. BMC Res Notes.

[88] Schmieder R, Edwards R (2011). Quality control and preprocessing of metagenomic datasets. Bioinformatics.

[89] Paterson AH, Bowers JE, Bruggmann R, Dubchak I, Grimwood J, Gundlach H (2009). The Sorghum bicolor genome and the diversification of grasses. Nature.

[90] Langmead B, Salzberg SL (2012). Fast gapped-read alignment with Bowtie 2. Nat Methods.

[91] Roberts A, Pachter L (2013). Streaming fragment assignment for real-time analysis of sequencing experiments. Nat Methods.

[92] Robinson MD, McCarthy DJ, Smyth GK (2010). edgeR: a Bioconductor package for differential expression analysis of digital gene expression data. Bioinformatics.

[93] Pfaffl MW. A new mathematical model for relative quantification in real-time RT-PCR. Nucleic Acids Research*.* 2001;29(9). 10.1093/nar/29.9.e45 PMC55695 11328886

[94] Notredame C, Higgins DG, Heringa J (2000). T-Coffee: a novel method for fast and accurate multiple sequence alignment. J Mol Biol.

[95] Hall TA (1999). BioEdit: a user-friendly biological sequence alignment editor and analysis program for Windows 95/98/NT. Nucleic Acids Symp Ser.

[96] Darriba D, Taboada GL, Doallo R, Posada D (2011). ProtTest 3: fast selection of best-fit models of protein evolution. Bioinformatics.

[97] Guindon S, Dufayard J-F, Lefort V, Anisimova M, Hordijk W, Gascuel O (2010). New algorithms and methods to estimate maximum-likelihood phylogenies: assessing the performance of PhyML 3.0. Syst Biol.

[98] Oakley AJ (2005). Glutathione transferases: new functions. Curr Opin Struct Biol.

[99] Faurobert M, Pelpoir E, Chaib J (2007). Phenol extraction of proteins for proteomic studies of recalcitrant plant tissues. Methods Mol Biol.

[100] Blomstedt CK, Gleadow RM, O'Donnell N, Naur P, Jensen K, Laursen T (2012). A combined biochemical screen and TILLING approach identifies mutations in Sorghum bicolor L. Moench resulting in acyanogenic forage production. Plant Biotechnol J.

