# Supplementary material for: Dhurrin metabolism in the developing grain of Sorghum bicolor (L.) Moench investigated by metabolite profiling and novel clustering analyses of time-resolved transcriptomic data
Source: BMC Genomics. 2016 Dec 13;17:1021. doi: 10.1186/s12864-016-3360-4 (PMC5154151; doi:10.1186/s12864-016-3360-4)
Supplement: Additional file 1: — A-F). Expression profile of CAS measured by qPCR, RNA integrity, concentration and purity, melting curve analysis, qPCR product run on 4% agarose gel, Sanger sequencing results of qPCR products and efficiency test of CAS, ZF and AAPK primers. (PDF 528 kb) [file 12864_2016_3360_MOESM1_ESM.pdf]

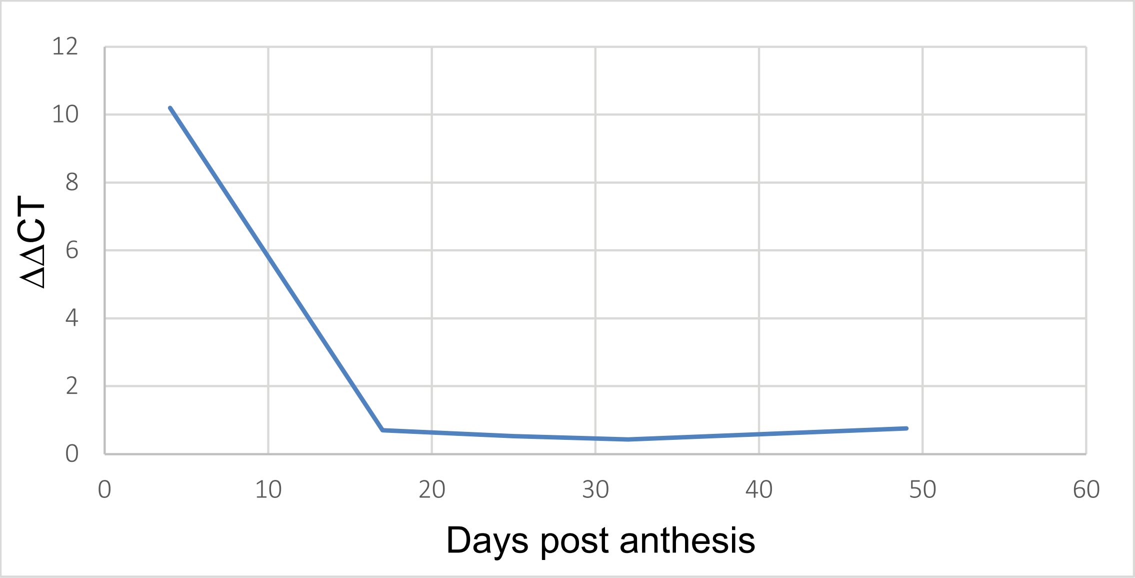

Additional file 1A. Expression profile of CAS measured by qPCR in the developing grains at day 4, 17, 25, 32 and 49 days after anthesis.

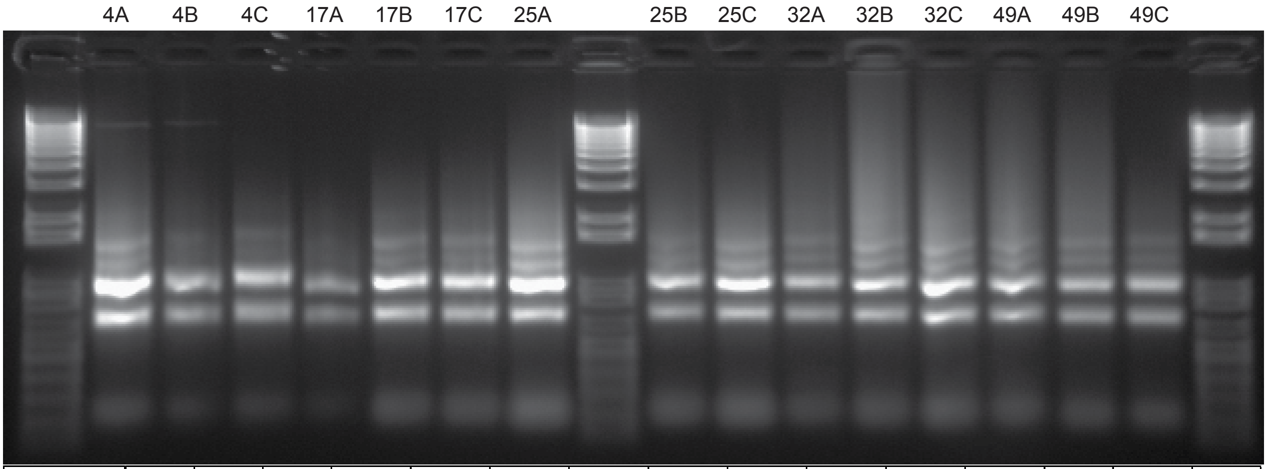

Sample	4A	4B	4C	17A	17B	17C	25A	25B	25C	32A	32B	32C	49A	49B	49C
ng/µL	154.20	416.20	315.70	1273.00	1999.00	1741.60	2924.80	3343.70	3482.60	1137.80	1537.60	1311.50	928.40	1352.00	627.80
260/280 ratio	2.02	2.06	2.09	2.09	2.10	2.10	2.06	2.01	2.00	2.10	2.08	2.08	2.05	2.05	2.04
260/230 ratio	2.23	2.20	2.34	2.46	2.43	2.40	2.26	2.18	2.13	2.30	2.25	2.20	2.10	2.06	2.05

Additional file 1B. The integrity, concentration and purity of the RNA used for the qPCR analysis, analyzed on a 1% agarose gel and with Nanodrop spectrophotometer (table below gel).

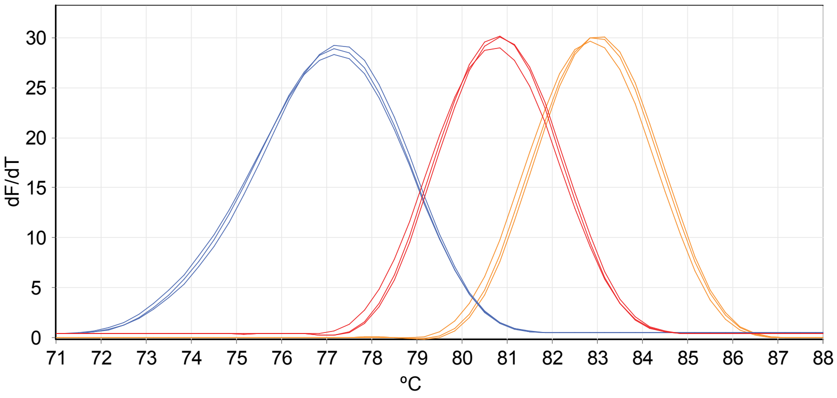

Additional file 1C. Melting curve analysis for the three genes used for the qPCR analysis. AAPK (blue), ZF (red), CAS (orange).

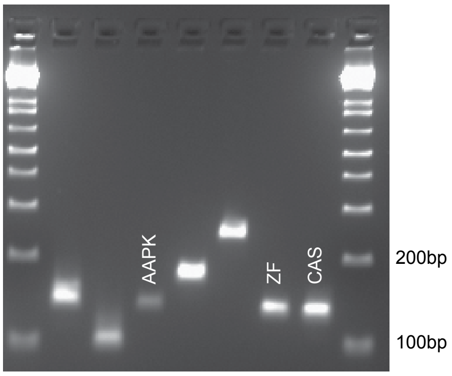

Additional file 1D. qPCR product run on 4% agarose gel and visualized with GelRed™. The size of the products corresponds to the DNA sequence they are supposed to replicate: AAPK (140bp), ZF (132bp) and CAS (133bp).

```
CAS         CATTGTGACTGTTCTTCCAAGCCTTGGGGAGCGATACTTGTCATCTGCGCTGTTTGATGAGTTGAGAAAGGAGGCTGAAGCAATGGAACCAGTGCCGGTG
Consensus                                         |||| |||||||||||||||||||||||||||||||||||||||||||||||||||||||||
Sequencing  ~~~~~~~~~~~~~~~~~~~~~~~~~~~~~~~~~~~~~~TGTCCTCTGCGCTGTTTGATGAGTTGAGAAAGGAGGCTGAAGCAATGGAACCAGTGCCGGTG
            GATTAATTTGGCCAATCAAACCGGGCACTGGAA
            ||||||||||||||||||||||||||||| | |
            GATTAATTTGGCCAATCAAACCGGGCACTAGGA

ZF          TCCACCAGCACTCAGGTTTCATTGAGCGCTGTAGTTTGTCCTAACTGATATGGTGTTGCATTGAGAAGGACTATTTGGAAACATGAGTTAAACTGCATCT
Consensus                                      ||||| || ||||||||||||||||||||||||||||||||||||||||||||||||||||||||
Sequencing  ~~~~~~~~~~~~~~~~~~~~~~~~~~~~~~~~~~~TTGTCGTA~CTGATATGGTGTTGCATTGAGAAGGACTATTTGGAAACATGAGTTAAACAGCATCT
            CTG~GTCCCTGGGATTCAAAAGTACCGAGTCCAGCT
            ||||||||||||||||||||||||||||||||||||
            CAGAGTCCCAGGGATTCAAAAGTACCGAGTCCAGCT

AAPK        GGAATGGTGCATTCATGCCGTGTTAATCCAGCAAATTCTG~TATTAGATTAATTAAGAAGGCTTCTGTTATGTTTATACTATGCCATTTCTTTGGAAGTT
Consensus                                    ||||||| | |||||||||||||||||||||||||||||||||||||||||||||||||||||||||
Sequencing  ~~~~~~~~~~~~~~~~~~~~~~~~~~~~~~~~CAATTCTGATGTTAGATTAATTAAGAAGGCTTCTGTTATGTTTATACTATGCCATTTCTTTGGAAGTT
            TGGATAGTTTGCTGATCAAATGCGTGGTAACCATTAGGAG
            ||||||||||||||||||||||||||||||||||||||||
            TGGATAGTTTGCTGATCAAATGCGTGGTAACCATTAGGAG
```

Additional file 1E. Alignment of Sanger sequencing result against the specific genes of interest CAS, ZF and AAPK. The primer binding sites for the forward and reverse primers are marked in red font.

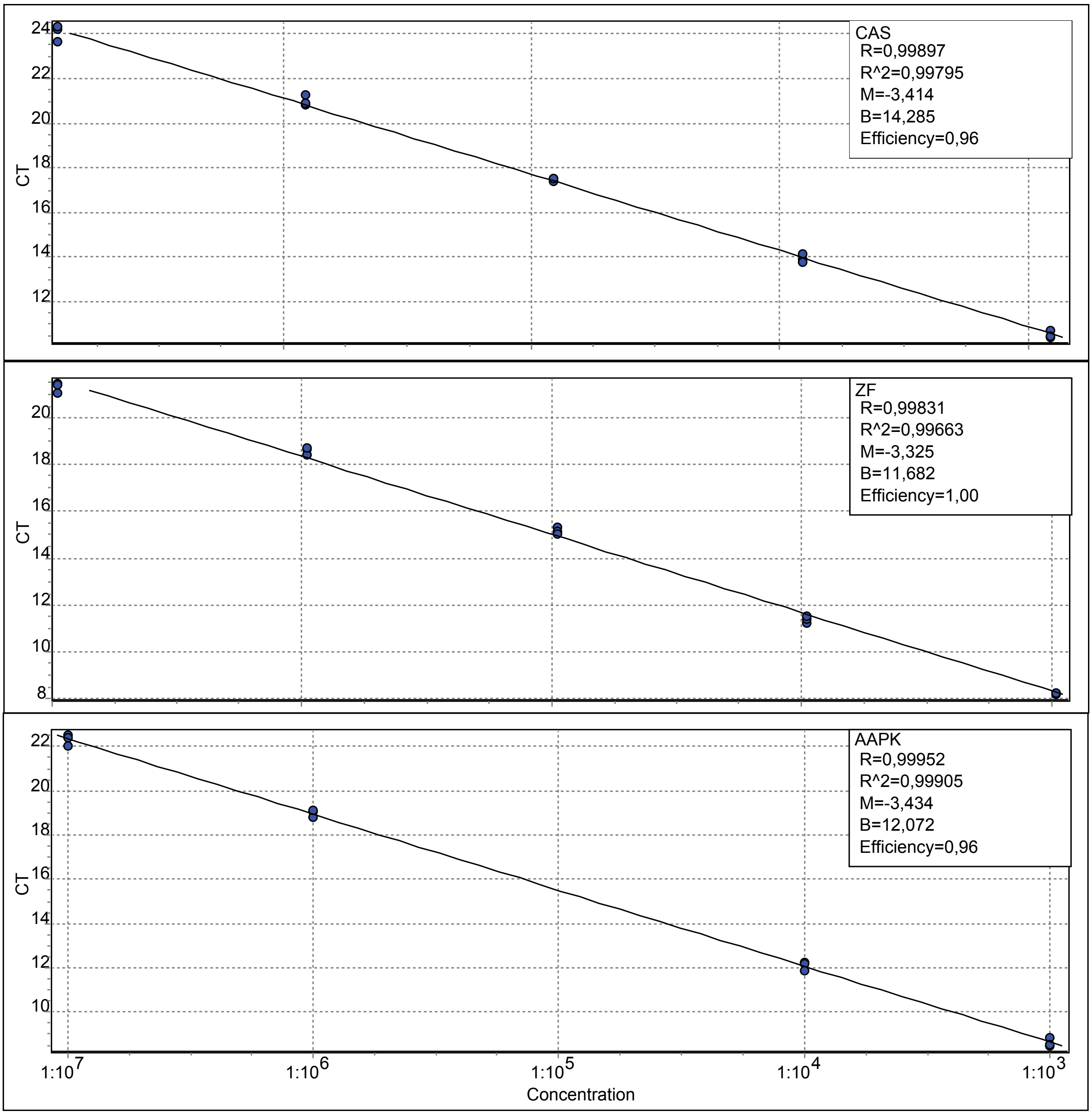

Additional file 1F. Efficiency test of the primers specific for the genes CAS, ZF and AAPK on serial dilutions of template in the following dilutions: 1:10^3, 1:10^4, 1:10^5, 1:10^6, 1:10^7.
