# Supplementary material for: Dhurrin metabolism in the developing grain of Sorghum bicolor (L.) Moench investigated by metabolite profiling and novel clustering analyses of time-resolved transcriptomic data
Source: BMC Genomics. 2016 Dec 13;17:1021. doi: 10.1186/s12864-016-3360-4 (PMC5154151; doi:10.1186/s12864-016-3360-4)
Supplement: Additional file 2: — Dendrogram showing specific clusters from the hierarchical clustering of all genes expressed in sorghum. (PDF 429 kb) [file 12864_2016_3360_MOESM2_ESM.pdf]

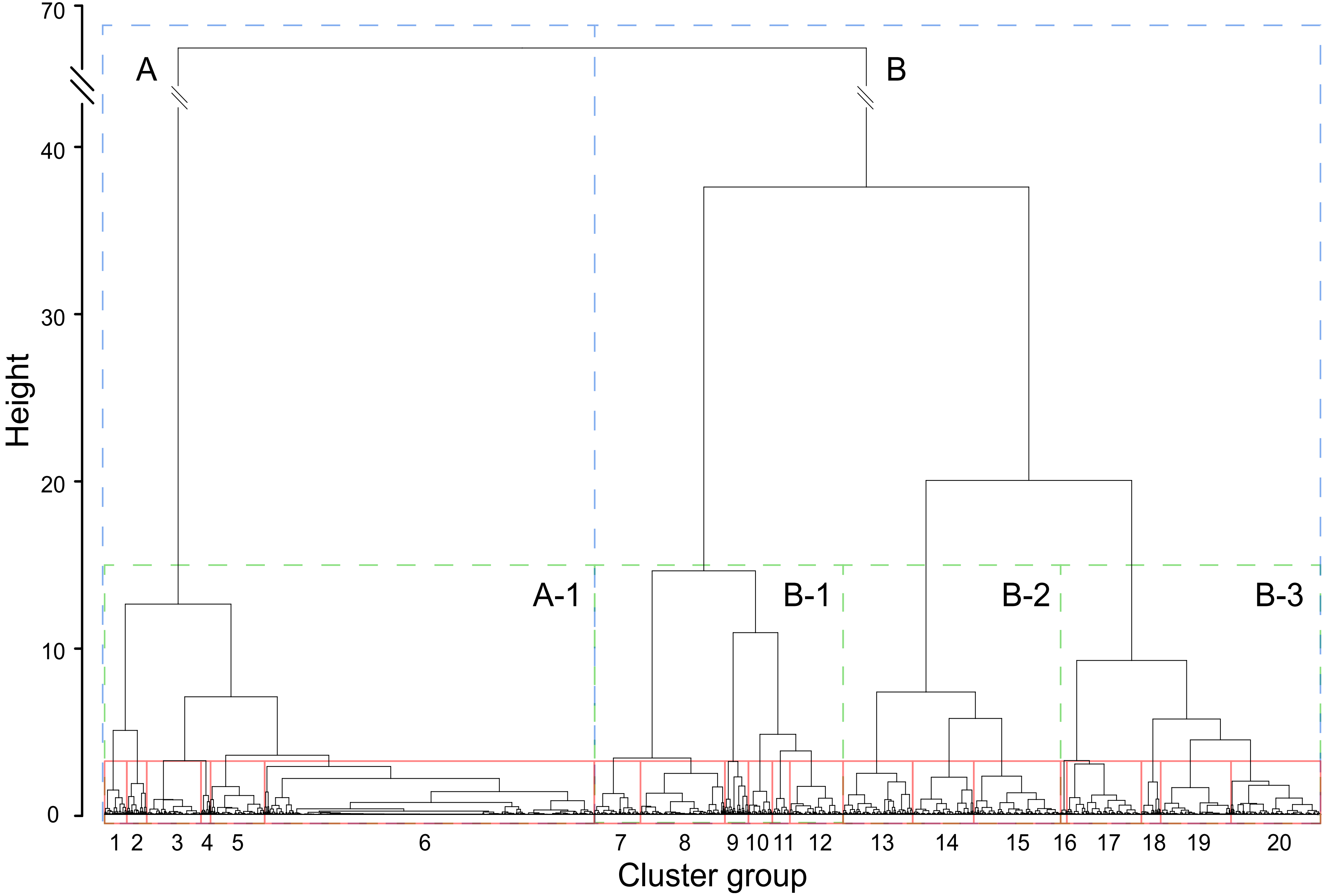

Additional file 2. Plot of the dendrogram from the hierarchical clustering, showing the relation between the 20 clusters marked by red boxes in the bottom of the figure. In the dendrogram, the cluster groups are divided into two separate clades denoted A and B. These are again divided into the sub-clades A-1, B-1, B-2 and B-3. In these subclades, the 20 clusters are contained.
