# Supplementary material for: Dhurrin metabolism in the developing grain of Sorghum bicolor (L.) Moench investigated by metabolite profiling and novel clustering analyses of time-resolved transcriptomic data
Source: BMC Genomics. 2016 Dec 13;17:1021. doi: 10.1186/s12864-016-3360-4 (PMC5154151; doi:10.1186/s12864-016-3360-4)
Supplement: Additional file 3: — Clustering analysis of transcript expression profiles divided into 20 groups of similar expression. (PDF 245 kb) [file 12864_2016_3360_MOESM3_ESM.pdf]

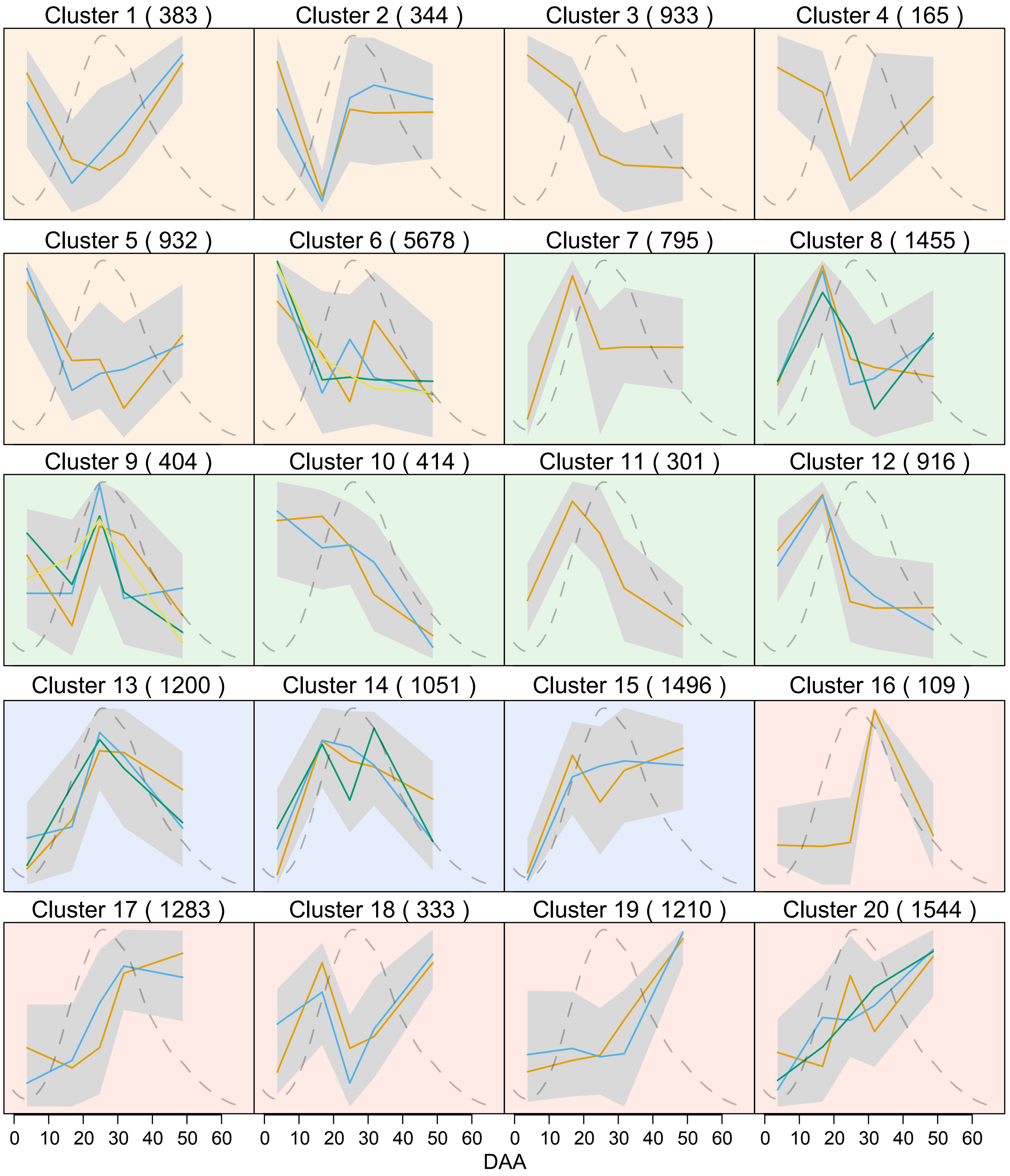

Additional file 3. Clustering analysis of transcript expression profiles. The hierarchical clustering is divided into 20 groups, which was further subdivided into 81 subgroups (colored lines in each cluster). These genes characterized genes investigated in this manuscript belong to the following cluster groups/subgroups: cytochrome P450 reductase (POR) 2A (Sobic.002G295100), CAS (Sobic.006G016900) and ACC oxidase (Sobic.003G197200) in 3-1; POR2C (Sobic.006G245400) in 5-2; POR2B (Sobic.007G088000) in 6-3; cytochrome P450 (CYP) 79A1 (Sobic.001G012300) and UDP-glycosyltransferase (UGT) 85B1 (Sobic.001G012400) in 8-1; nitrilase (NIT) 4B1 (Sobic.004G225000) in 10-1; CYP71E1 (Sobic.001G012200) in 12-1; HNL (Sobic.004G335500) in 13-1; NIT4B2 (Sobic.004G225100) in 15-2 and NIT4A (Sobic.004G225200) in 20-2. The number of genes in each cluster is listed in parenthesis next the cluster number. The grey field represents the span of all the expression profiles in a single cluster. The dashed line represents the concentration of dhurrin pr. mg tissue through the development in relation to the expression profiles.
