# Supplementary material for: Dhurrin metabolism in the developing grain of Sorghum bicolor (L.) Moench investigated by metabolite profiling and novel clustering analyses of time-resolved transcriptomic data
Source: BMC Genomics. 2016 Dec 13;17:1021. doi: 10.1186/s12864-016-3360-4 (PMC5154151; doi:10.1186/s12864-016-3360-4)
Supplement: Additional file 4: — A-C. Transcriptional and proteomic ratio between CYP71E1 and CYP79A1. The protein ratio is calculated from integrated areas of Western blot analysis of CYP79A1 and CYP71E1 using imageJ. (PDF 758 kb) [file 12864_2016_3360_MOESM4_ESM.pdf]

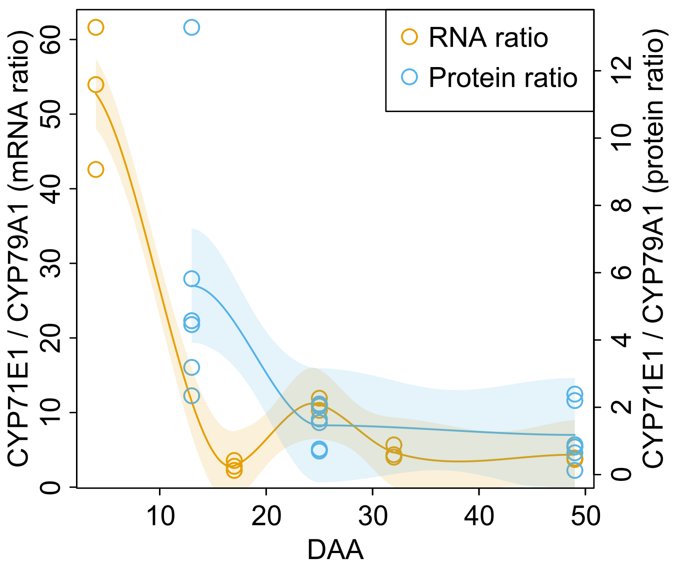

Additional file 4A. Transcriptional and proteomic ratio between CYP71E1 and CYP79A1. To compare the difference in transcript and enzyme levels between CYP71E1 and CYP79A1, the values measured were converted to ratios. The insert display normalized data to better visualize the trend of the transcript and enzyme ratios. The standard deviation for the transcripts are based on three independent biological replicates. For the enzyme ratios, the standard deviation represent technical variation and the three different lines three different western blots. In the table, the values obtained by analyzing the blots with ImageJ (as described in Materials and methods) are displayed.

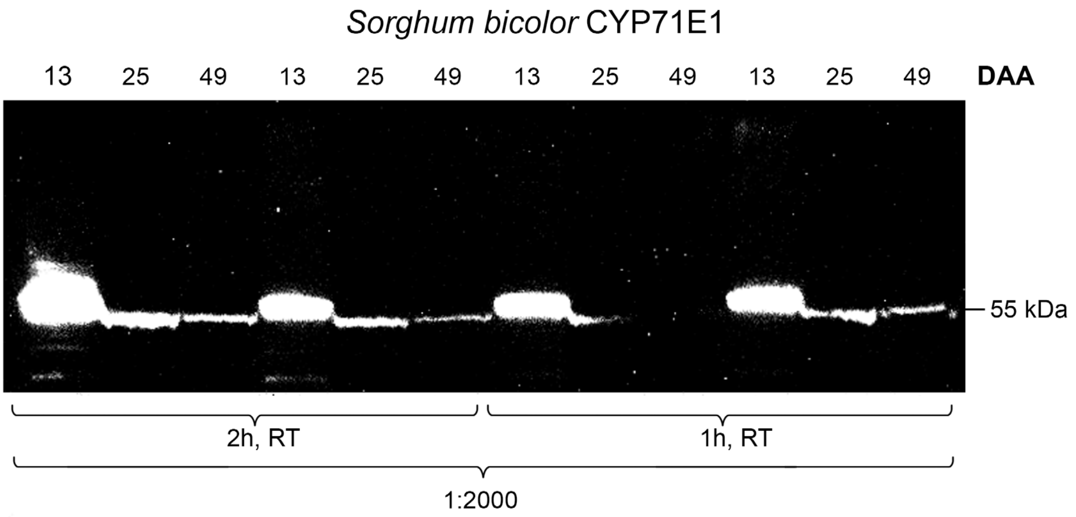

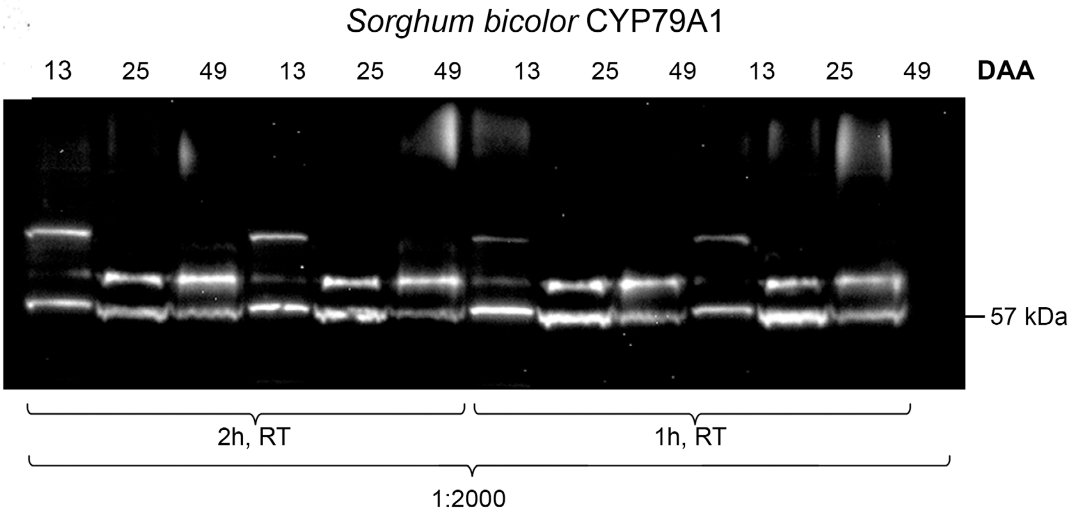

Additional file 4B. Result of western blot analysis of total protein extraction with antibodies against CYP79A1 and CYP71E1. In this run the total protein were mixed with sample buffer and heated at 60°C for 15min. An antibody dilution of 1:1500 were used. The size of CYP79A1 (57 kDa) and CYP71E1 (55 kDa) are marked on each blot.

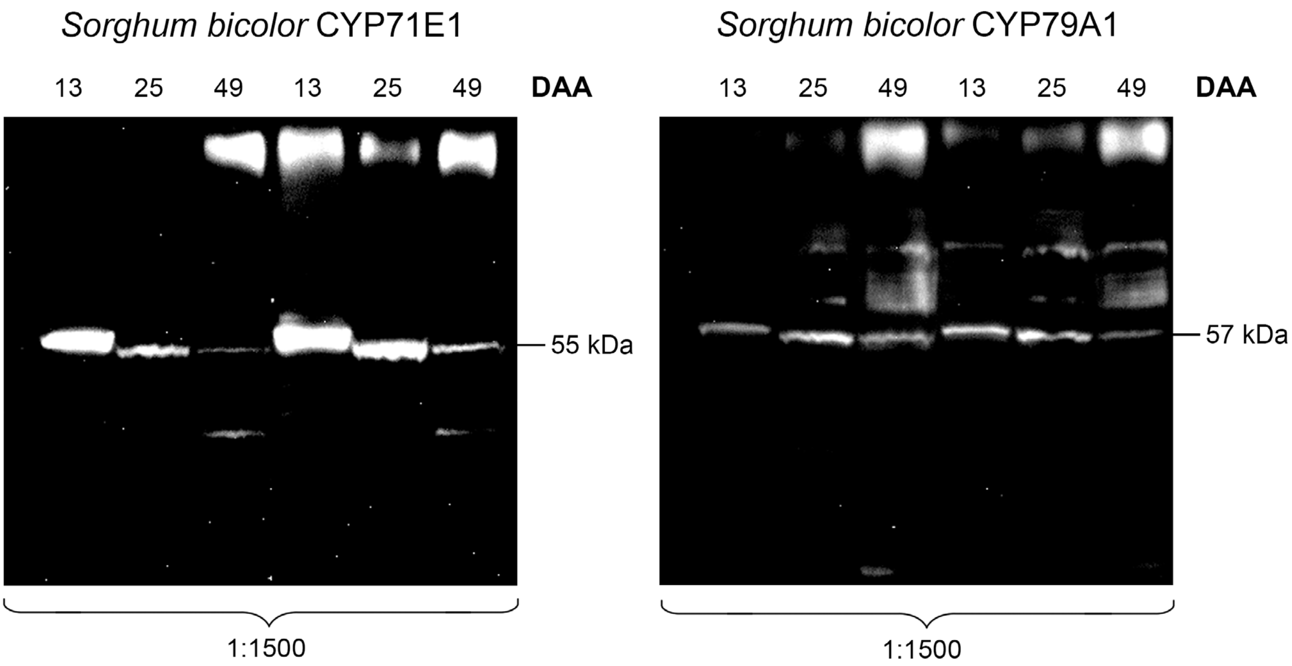

Additional file 4C. Result of western blot analysis of total protein extraction with antibodies against CYP79A1 and CYP71E1. In this run the total protein were mixed with sample buffer and heated at 60°C or 40°C for 15min. An antibody dilution of 1:2000 were used. The size of CYP79A1 (57 kDa) and CYP71E1 (55 kDa) are marked on each blot.

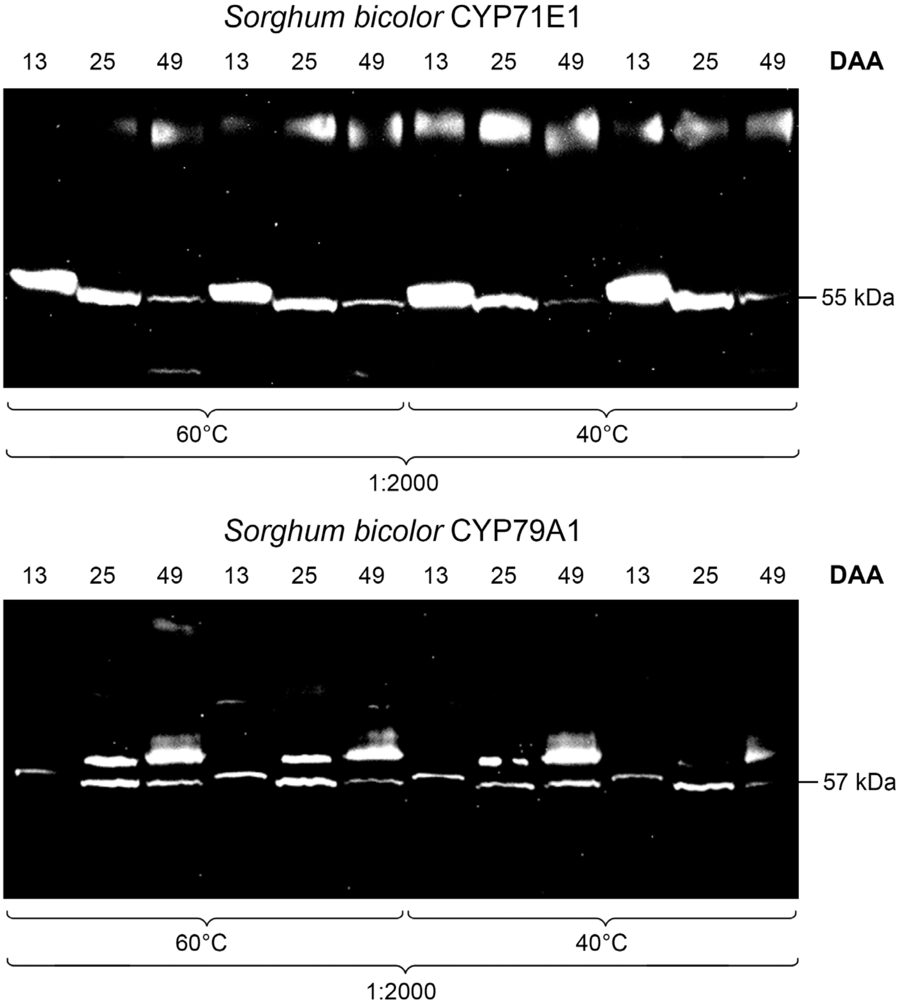

Additional file 4D. Result of western blot analysis of total protein extraction with antibodies against CYP79A1 and CYP71E1. In this run the total protein were mixed with sample buffer and incubated at RT for either 1 or 2h. An antibody dilution of 1:2000 were used. The size of CYP79A1 (57 kDa) and CYP71E1 (55 kDa) are marked on each blot.
