# Supplementary material for: Dhurrin metabolism in the developing grain of Sorghum bicolor (L.) Moench investigated by metabolite profiling and novel clustering analyses of time-resolved transcriptomic data
Source: BMC Genomics. 2016 Dec 13;17:1021. doi: 10.1186/s12864-016-3360-4 (PMC5154151; doi:10.1186/s12864-016-3360-4)
Supplement: Additional file 7: — Consensus sequences around the EKC catalytic triad of the nitrilase superfamily defined by genes found in sorghum. (PDF 231 kb) [file 12864_2016_3360_MOESM7_ESM.pdf]

Nitrilase	F	P	E	a	F	H	R	K	l	.	P	T	L	.	C	W	E	N	r	m	P
β-Ureidoproprionase	l	Q	E	A	W	h	R	K	N	H	I	P	N	I	C	Y	G	R	h	H	P
Glutamine-dependent NAD synthetase	G	P	E	L	E	P	P	K	M	.	L	A	E	t	C	E	E	L	f	t	.
Nit	l	P	E	.	w	.	R	K	.	H	L	F	.	.	C	.	D	.	R	F	.
N-carbamyl putrescine amidohydrolase	i	Q	E	L	F	Y	R	K	s	H	L	P	g	I	C	W	D	Q	W	F	P

Additional file 7. Consensus sequences around EKC catalytic triad. The catalytic residues are marked by red. Uppercase letters represents residues in accordance with the residues specified in Pace and Brenner (2001) and lowercase letters, represents residues with internal consensus, but not with the former article. The dots represents varying residues.
