# Supplementary material for: Dhurrin metabolism in the developing grain of Sorghum bicolor (L.) Moench investigated by metabolite profiling and novel clustering analyses of time-resolved transcriptomic data
Source: BMC Genomics. 2016 Dec 13;17:1021. doi: 10.1186/s12864-016-3360-4 (PMC5154151; doi:10.1186/s12864-016-3360-4)
Supplement: Additional file 8: — Dendrogram showing specific clusters from the hierarchical clustering of expressed UGTs in sorghum. (PDF 153 kb) [file 12864_2016_3360_MOESM8_ESM.pdf]

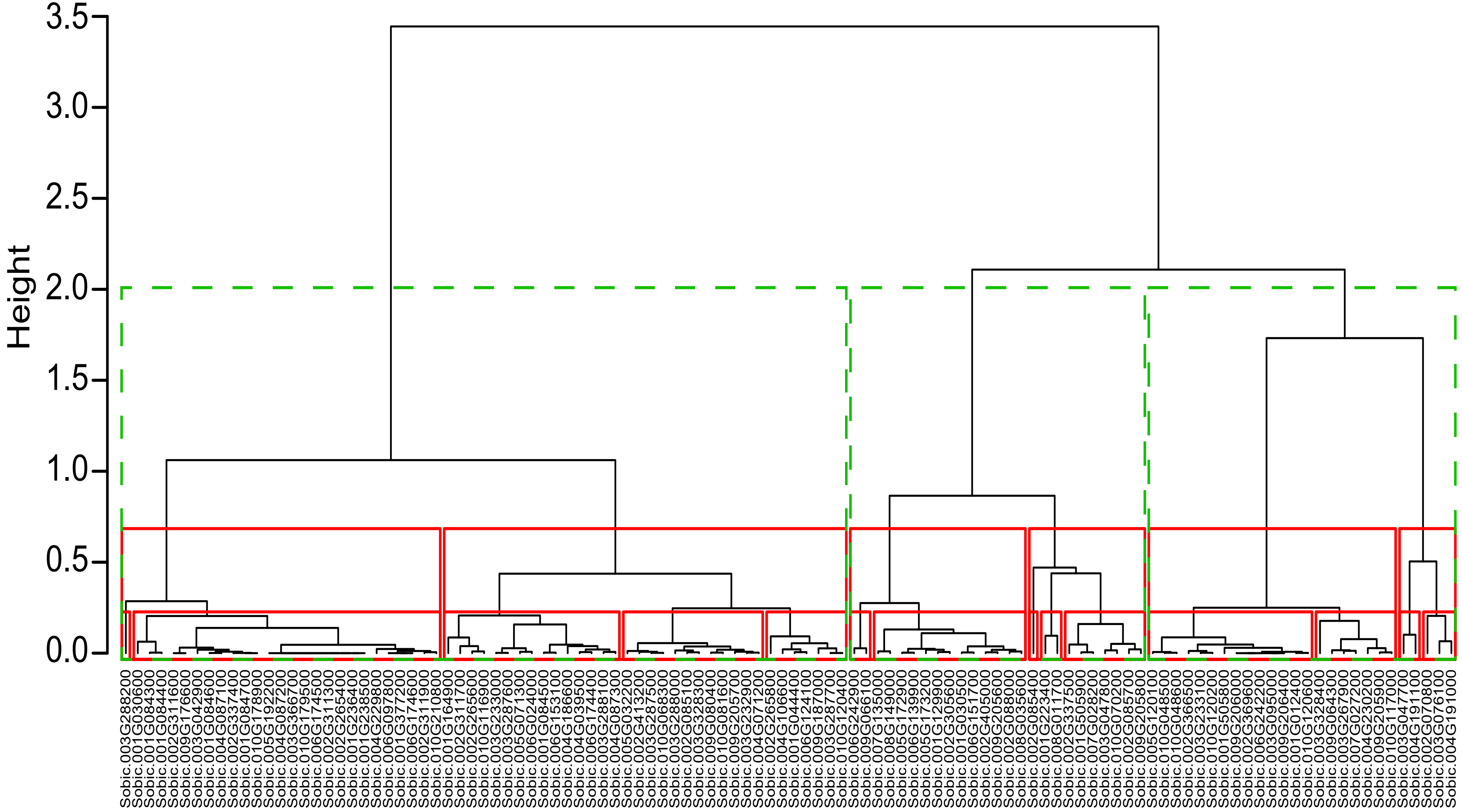

Additional file 8. Plot of the dendrogram from the hierarchical clustering of all UGT genes, showing the relation between the 6 clusters and the 14 subgroups marked by red boxes in the bottom of the figure. The gene names belonging to the different clusters are listed below. The 6 clusters can be divided into 3 clades (marked by green dashed lines) containing genes with an overall similar profile.
