# Supplementary material for: Dhurrin metabolism in the developing grain of Sorghum bicolor (L.) Moench investigated by metabolite profiling and novel clustering analyses of time-resolved transcriptomic data
Source: BMC Genomics. 2016 Dec 13;17:1021. doi: 10.1186/s12864-016-3360-4 (PMC5154151; doi:10.1186/s12864-016-3360-4)
Supplement: Additional file 9: — A-L. Phylogenetic trees for the genes involved in flavonoid metabolism. (PDF 347 kb) [file 12864_2016_3360_MOESM9_ESM.pdf]

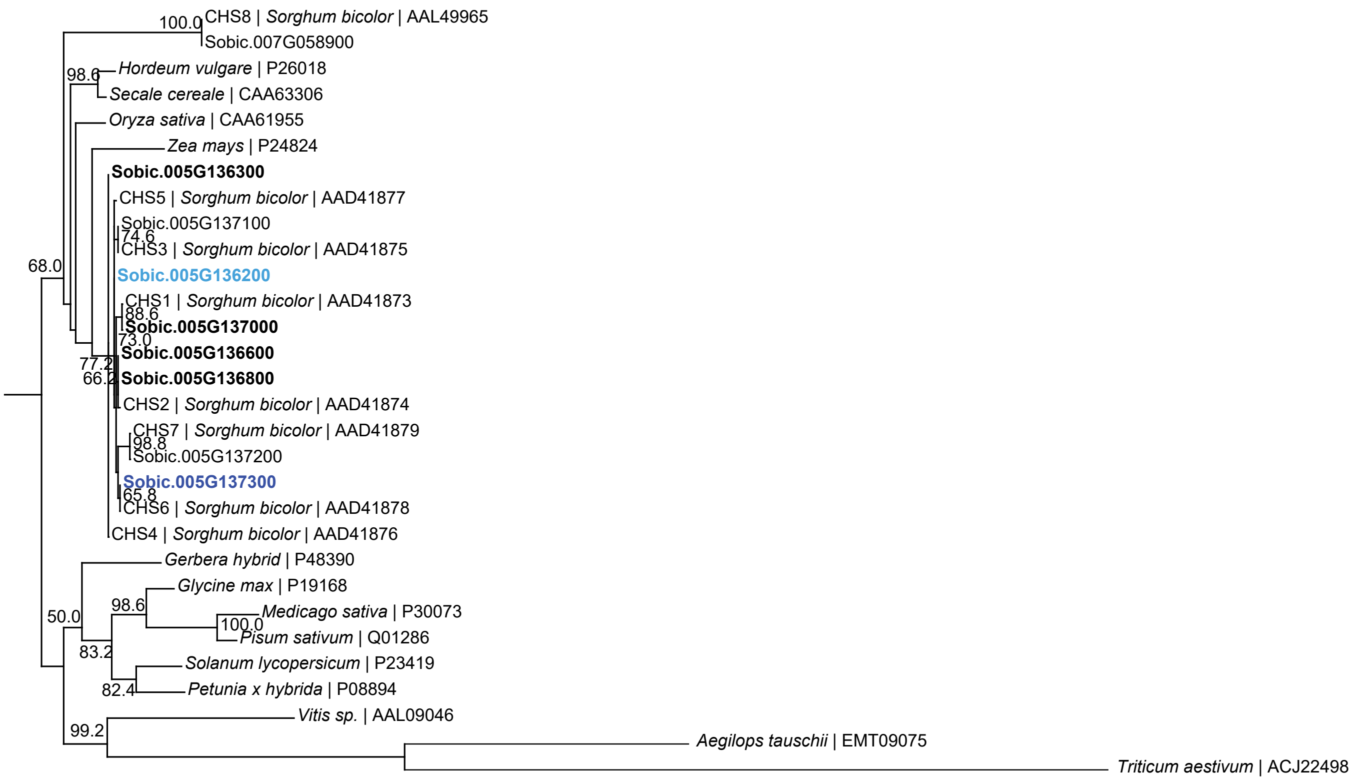

Additional file 9A. Phylogenetic tree of chalcone synthase (CHS). The tree is rerooted to divide the tree into a monocot and dicot clade. The bootstrapping values in percent are generated from 500 replicates, with only values above 50% shown. Genes expressed in the developing grains are shown in bold font with the colors relating to the profiles in Fig. 12.

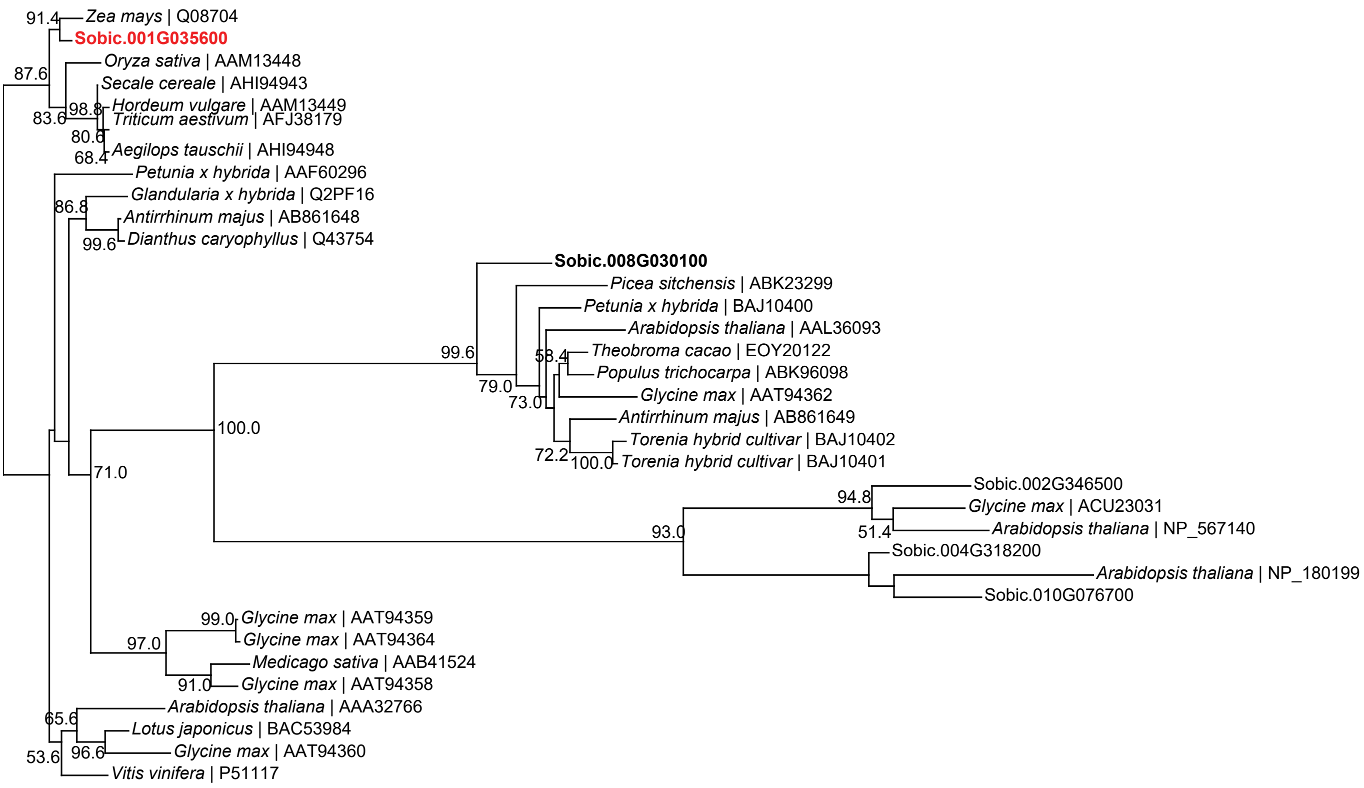

Additional file 9B. Phylogenetic tree of chalcone isomerase (CHI). The tree is constructed in a similar manner to Additional file 10A. The tree is rerooted to divide the tree into a monocot and dicot clade.

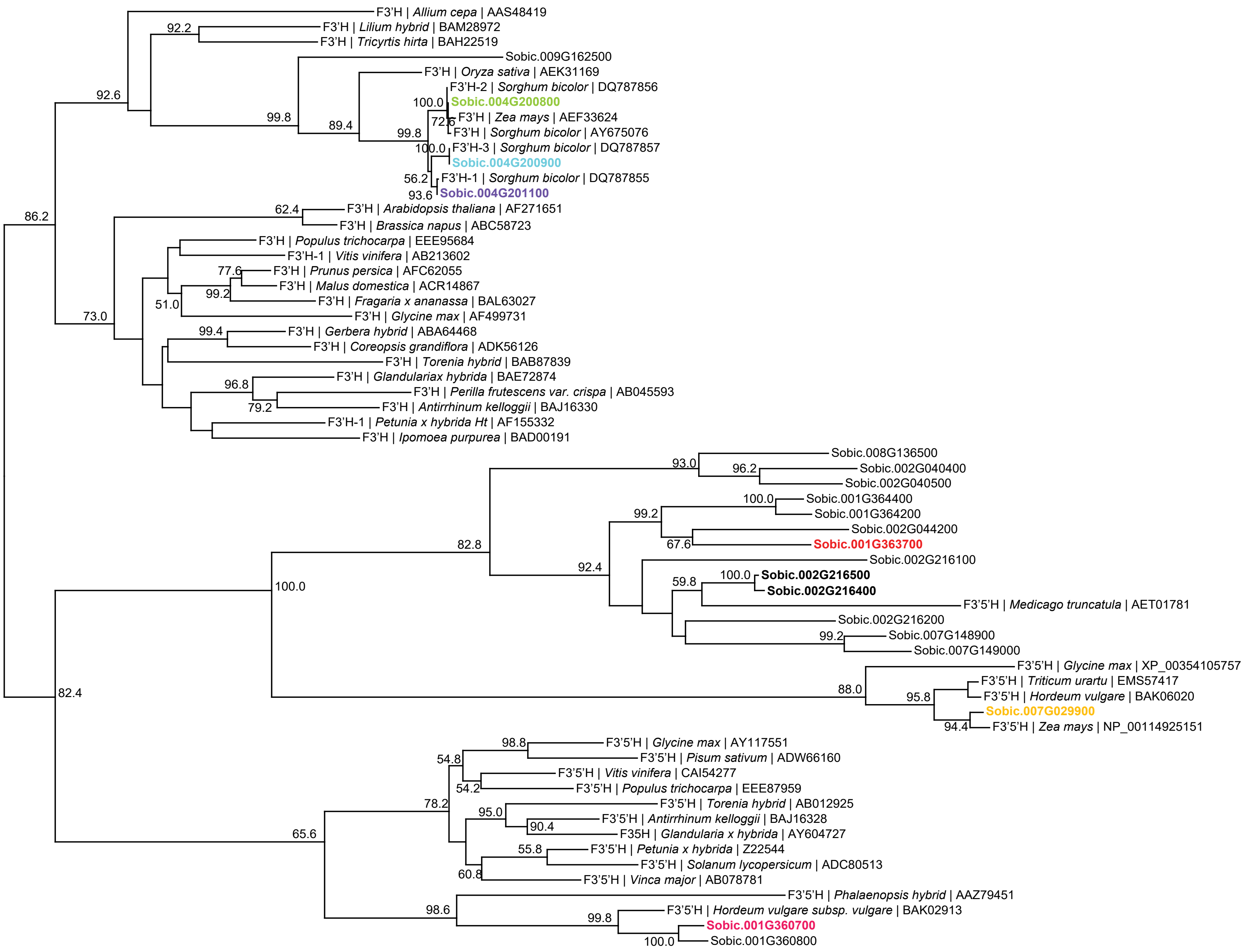

Additional file 9C. Phylogenetic tree of flavonoid 3'-hydroxylase (F3'H) and flavonoid 3',5'-hydroxylase (F3'5'H). The tree is constructed in a similar manner to Additional file 10A. The tree is divided into two clades containing the F3'H or F3'5'H genes. Each of these clades are further subdivided into a monocot or dicot clade.

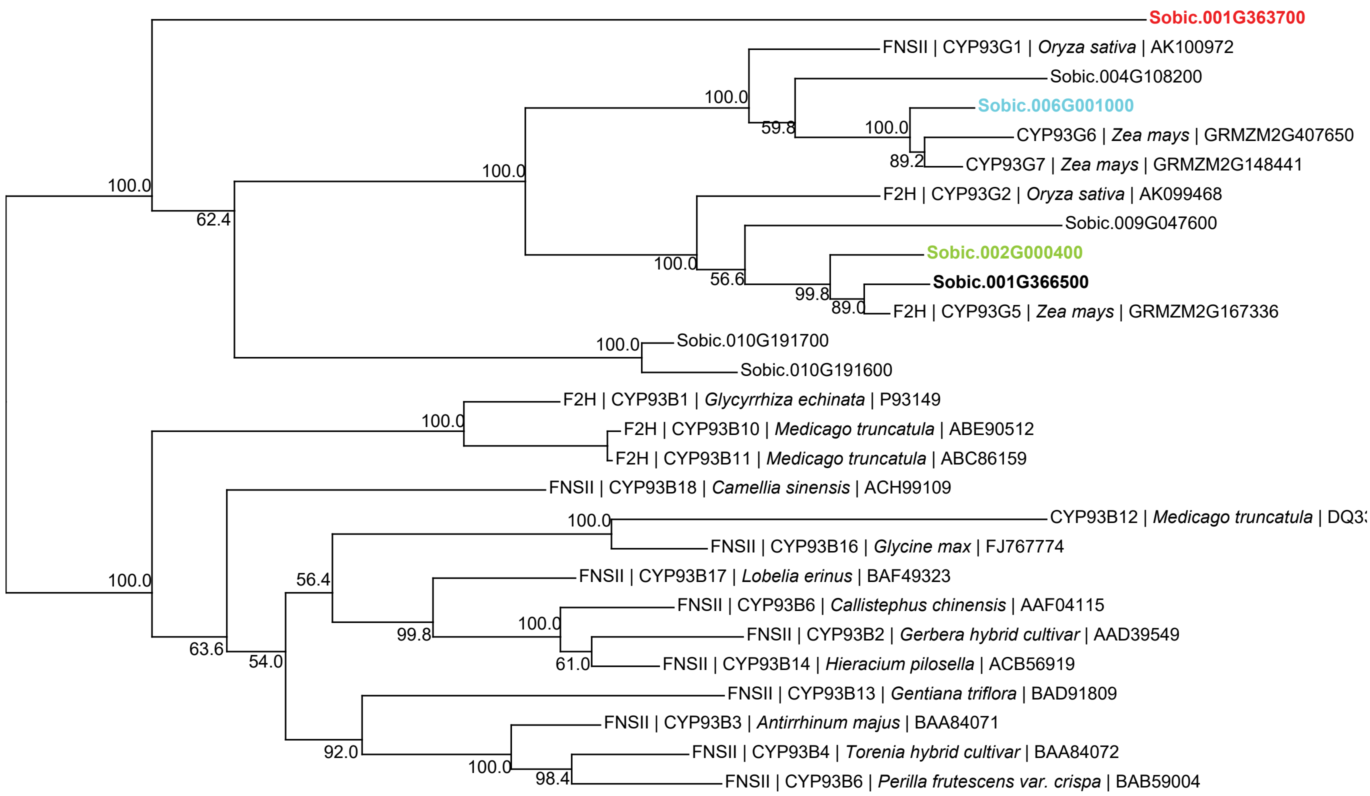

Additional file 9D. Phylogenetic tree of flavone synthase II (FNSII) and (2S)-flavanone 2-hydroxylase (F2H). The tree is constructed in a similar manner to Additional file 10A. The tree is rerooted to divide the tree into a monocot and dicot clade, which each of these two clades separated into a FNS and a F2H clade.

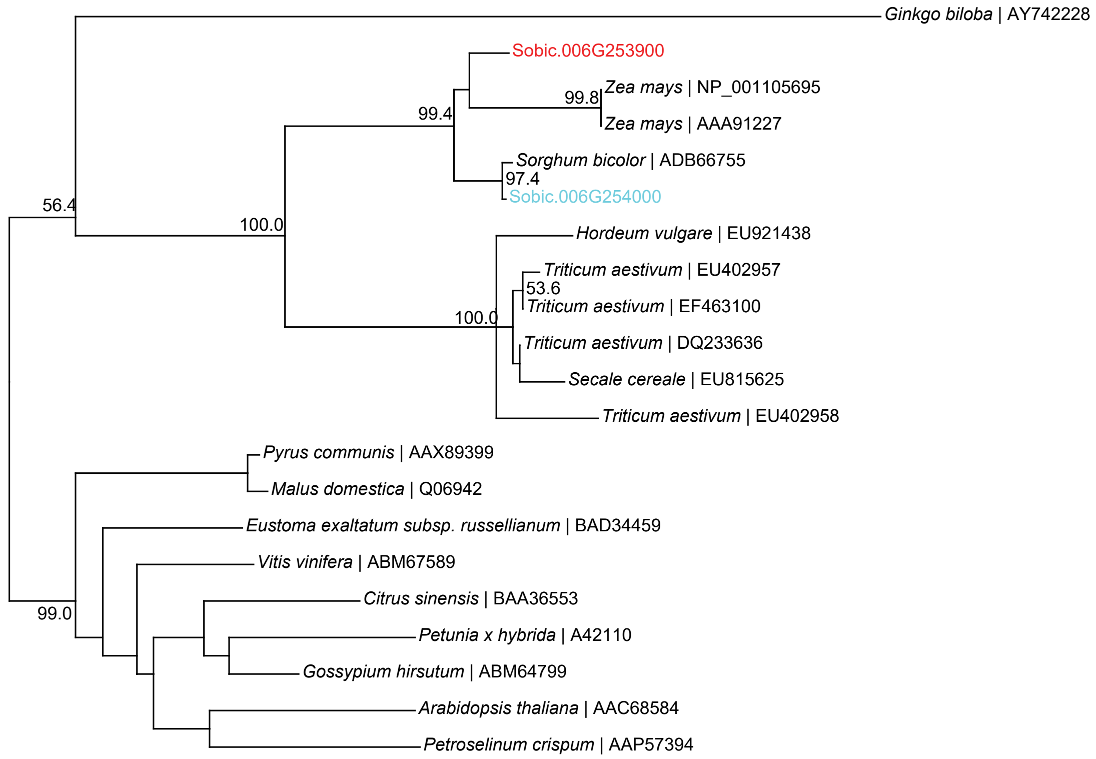

Additional file 9E. Phylogenetic tree of flavanone 3-hydroxylase (F3H). The tree is constructed in a similar manner to Additional file 10A. The tree is rerooted to divide the tree into a monocot and dicot clade.

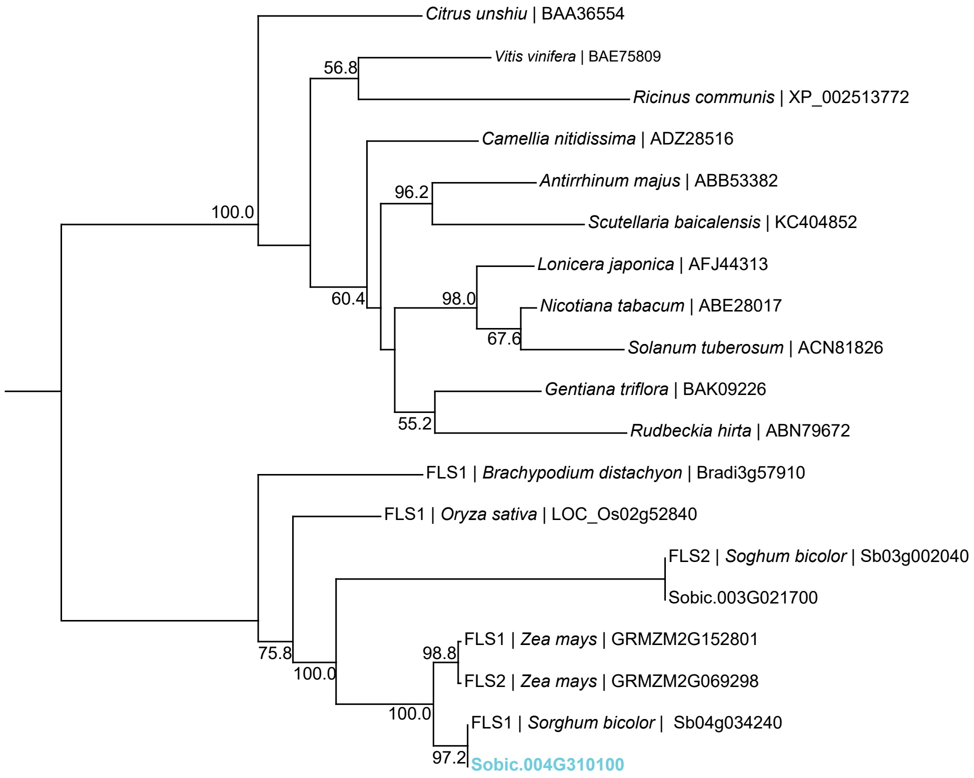

Additional file 9F. Phylogenetic tree of flavonol synthases (FLS). The tree is constructed in a similar manner to Additional file 10A. The tree is rerooted to divide the tree into a monocot and dicot clade.

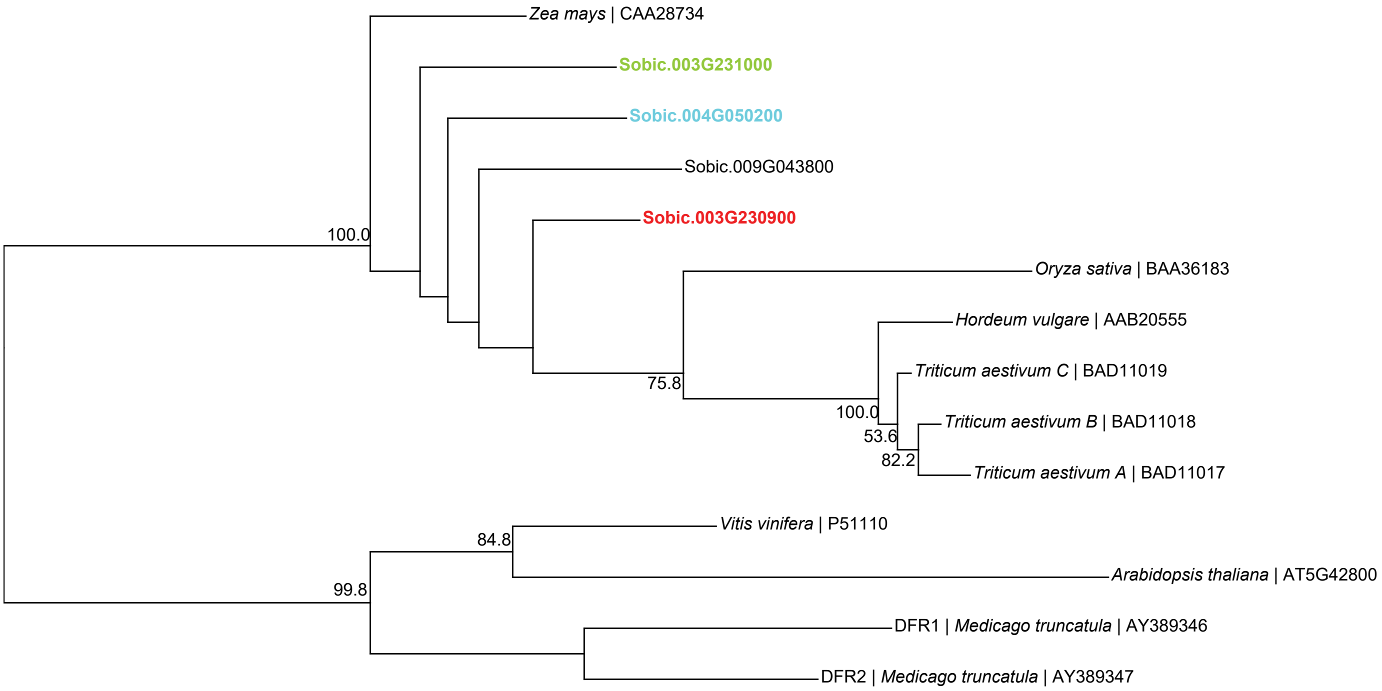

Additional file 9G. Phylogenetic tree of dihydroflavonol reductase (DFR). The tree is constructed in a similar manner to Additional file 10A. The tree is rerooted to divide the tree into a monocot and dicot clade.

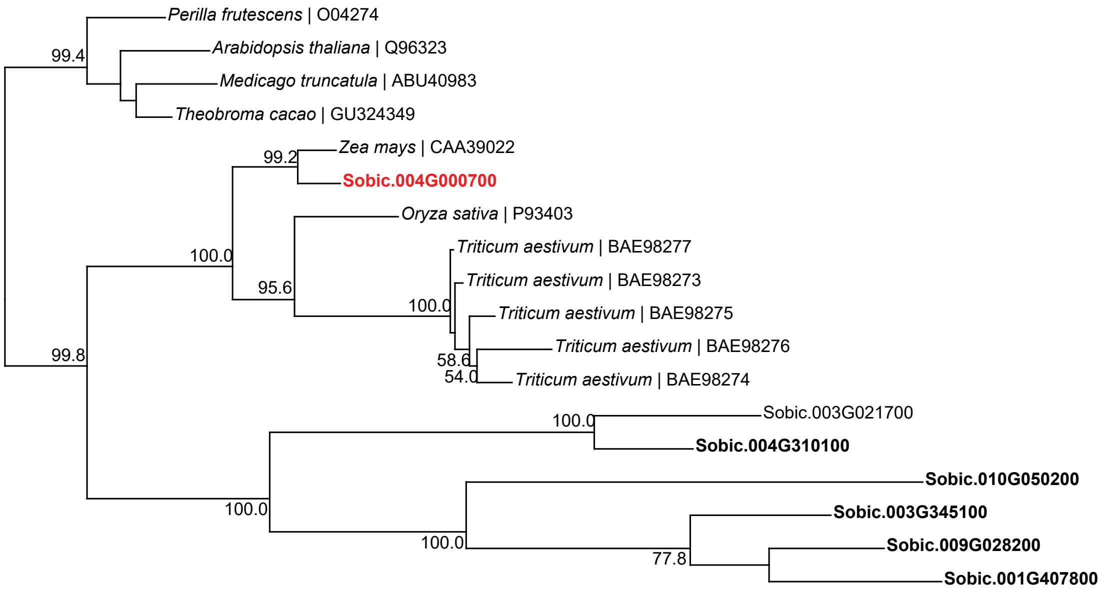

Additional file 9H. Phylogenetic tree of anthocyanidin synthase (ANS). The tree is constructed in a similar manner to Additional file 10A.The tree is rerooted to divide the tree into a monocot and dicot clade.

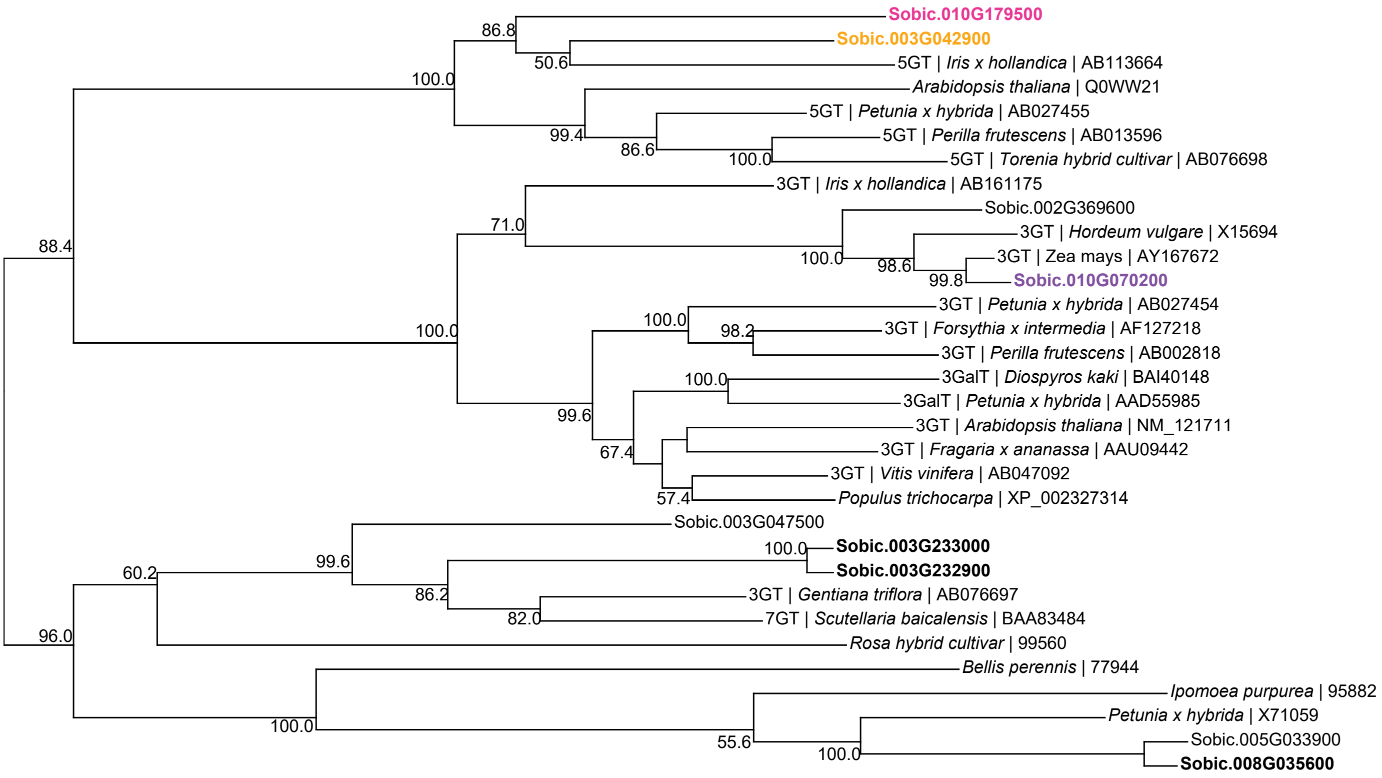

Additional file 9I. Phylogenetic tree of glycosyltransferase (GT). The tree is rerooted into a clade containing 5GT and 3GT genes and another clade containing more unspecific GT genes. Within the 3GT and 5GT clade, the genes is further grouped into monocot and dicot clades.

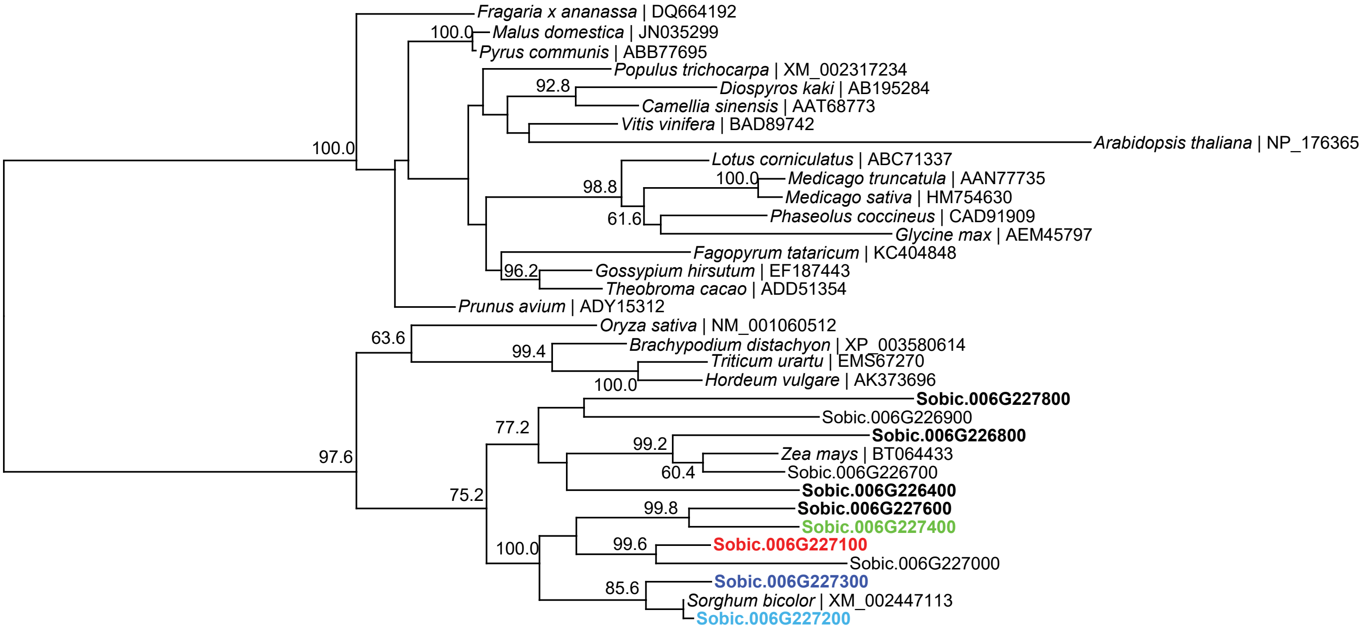

Additional file 9J. Phylogenetic tree of anthocyanidin reductase (ANR). The tree is constructed in a similar manner to Additional file 10A. The tree is rerooted to divide the tree into a monocot and dicot clade.

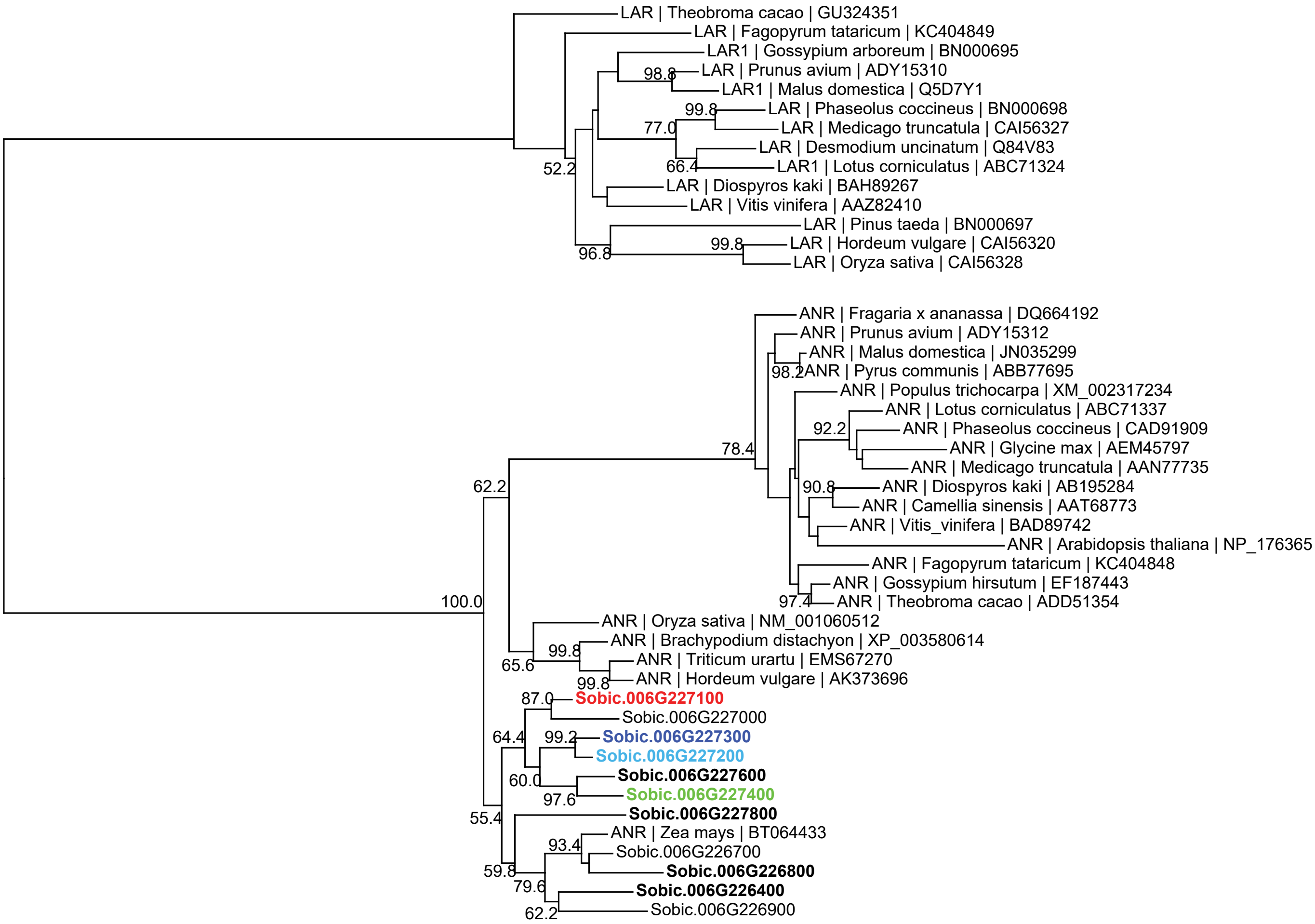

Additional file 9K. Phylogenetic tree of leucoanthocyanidin reductase (LAR) and anthocyanidin reductase (ANR). The tree is constructed in a similar manner to Additional file 10A. The LAR and ANR genes are clearly separated into two distinct clades. In the ANR clade, the genes are grouped into monocot and dicot clades, while the separation is less clear in the LAR clade.

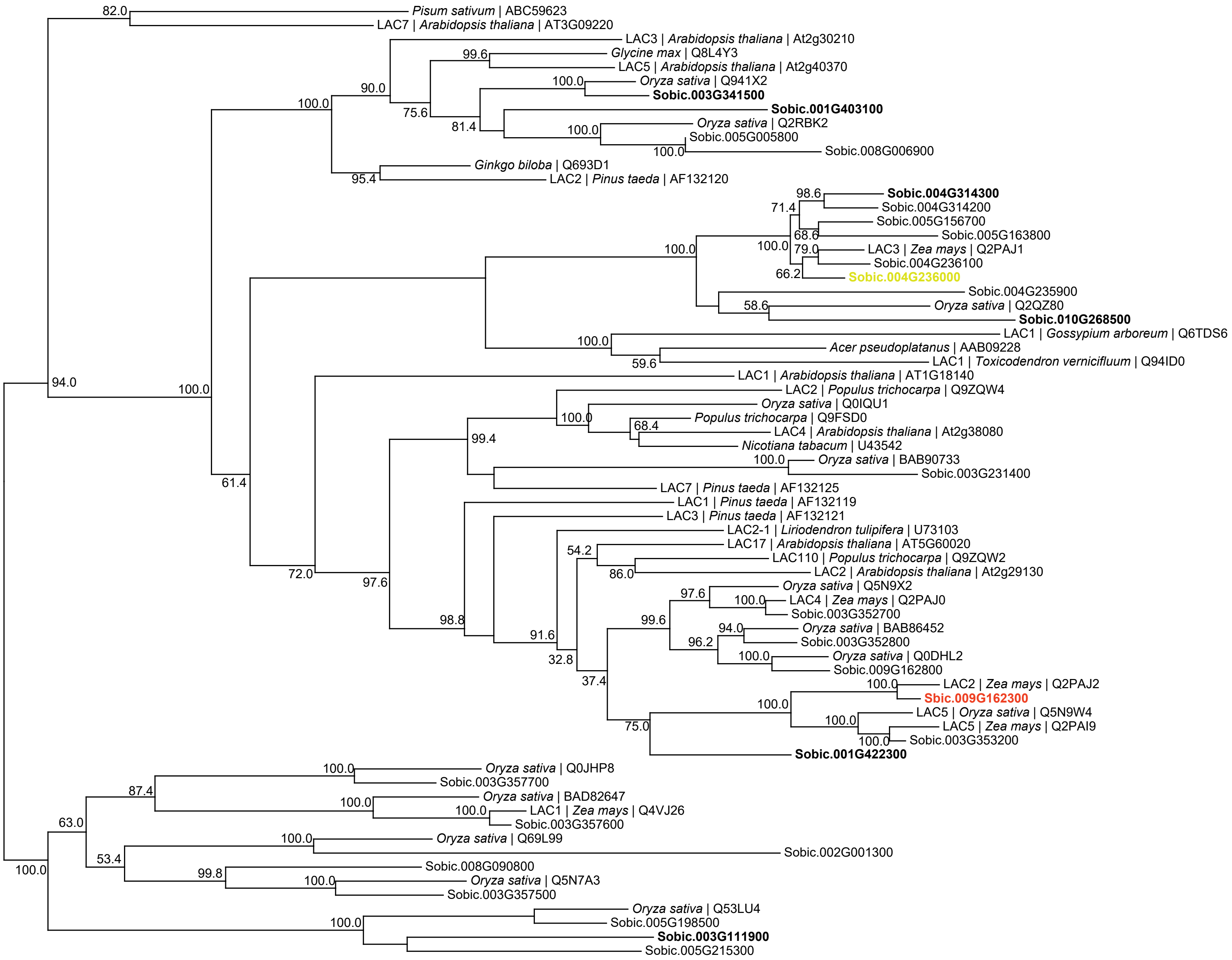

Additional file 9L. Phylogenetic tree of laccase (LAC). The tree is constructed in a similar manner to Additional file 10A.
