# Supplementary material for: Dhurrin metabolism in the developing grain of Sorghum bicolor (L.) Moench investigated by metabolite profiling and novel clustering analyses of time-resolved transcriptomic data
Source: BMC Genomics. 2016 Dec 13;17:1021. doi: 10.1186/s12864-016-3360-4 (PMC5154151; doi:10.1186/s12864-016-3360-4)
Supplement: Additional file 10: — Expression profiles for genes involved in the flavonoid biosynthesis in developing sorghum grain. (PDF 657 kb) [file 12864_2016_3360_MOESM10_ESM.pdf]

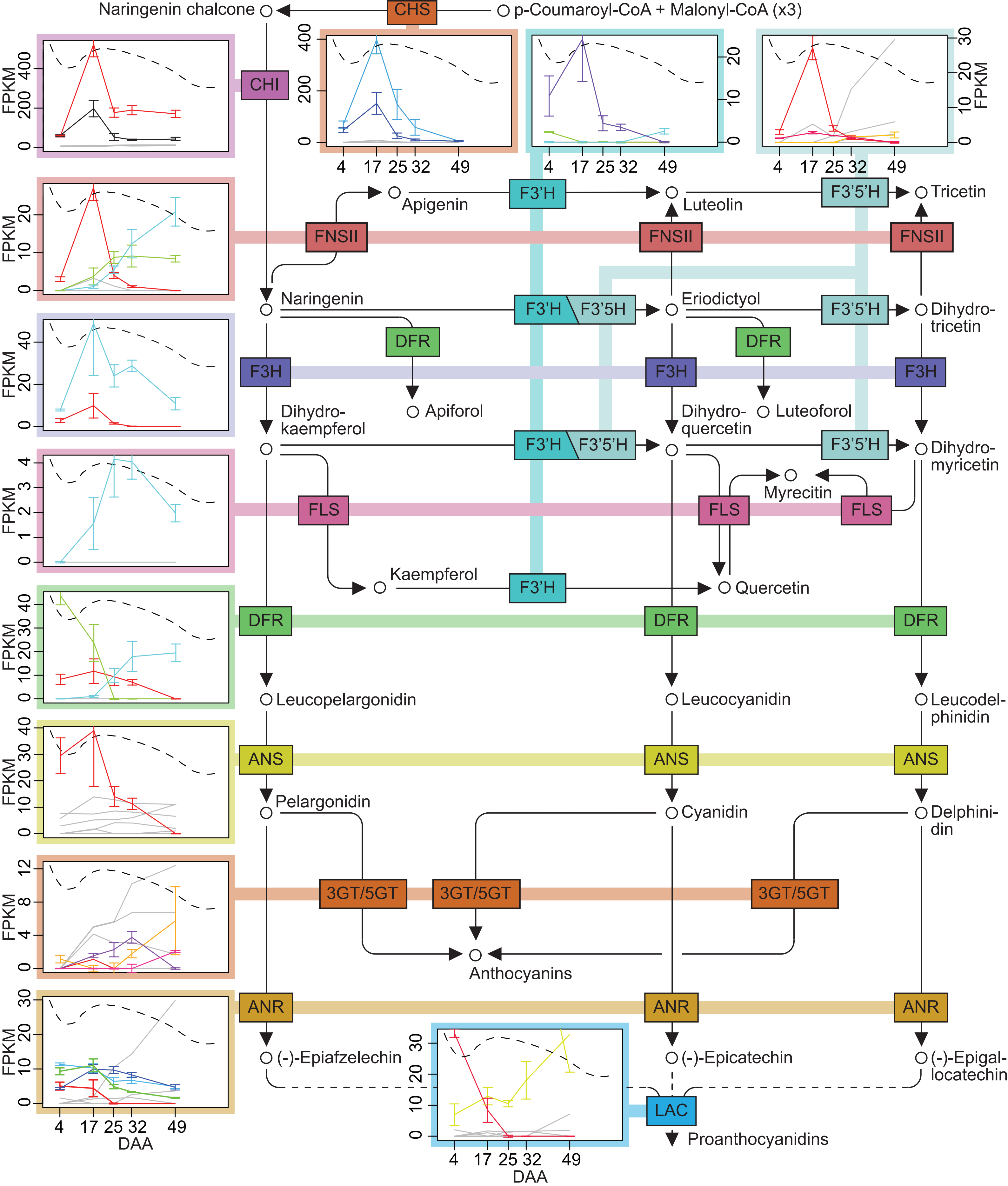

Additional file 10. Expression profiles for genes involved in the flavonoid biosynthesis in developing sorghum grain. The dashed line represents the PA absorbance pr. mg grain tissue (Fig. 1B). Transcript abundance is shown in FPKM. The colors of individual expression profiles relate to the genes in the Additional file 9A-L.
